# Intestinal epithelial *Ceacam1* deficiency prevents steroid-refractory acute gut graft-versus-host disease

**DOI:** 10.1172/jci.insight.186984

**Published:** 2025-09-09

**Authors:** Qingxiao Song, Moqian Zheng, Qinjian Li, Xiwei Wu, Boxi Lin, Tae Hyuk Kang, Hanjun Qin, Maciej Kujawski, Raju K. Pillai, James L. Lin, Ryotaro Nakamura, John Shively, Paul J. Martin, Defu Zeng

**Affiliations:** 1 Arthur D. Riggs Diabetes and Metabolism Research Institute, The Beckman Research Institute, and; 2 Hematologic Malignancies and Stem Cell Transplantation Institute, City of Hope National Medical Center, Duarte, California, USA.; 3 Medical Center of Hematology, Xinqiao Hospital, Chongqing, China.; 4 Jinfeng Laboratory, Chongqing, China.; 5 Department of Integrative Genomics Core, The Beckman Research Institute of City of Hope, Duarte, California, USA.; 6 Department of Clinical Laboratory, People’s Hospital Affiliated to Fujian Traditional Chinese Medicine University, Fuzhou, China.; 7 Department of Pathology and; 8 Department of Gastroenterology, City of Hope National Comprehensive Cancer Center, Duarte, California, USA.; 9 Fred Hutchinson Cancer Research Center, University of Washington, Seattle, Washington, USA.

**Keywords:** Hematology, Immunology, Transplantation, Bone marrow transplantation, Cellular immune response, Mouse models

## Abstract

Steroid-refractory gut acute graft-versus-host disease (SR-Gut-aGVHD) is the major cause of nonrelapse death after allogeneic hematopoietic cell transplantation. High numbers of donor-type IL-22^+^ T cells, IL-22–dependent dysbiosis, and loss of antiinflammatory CX3CR1^hi^ mononuclear phagocytes (MNPs) play critical roles in SR-Gut-aGVHD pathogenesis. CEACAM1 on intestinal epithelial cells (IECs) is proposed to regulate bacterial translocation and subsequent immune responses in the intestine. Here, with imaging mass cytometry (IMC), combined scRNA-Seq with ATAC-Seq, and high-dimensional flow cytometry analysis, we show that CEACAM1 expression was enhanced on IECs in murine and human SR-Gut-aGVHD. *Ceacam1* deficiency on host IECs effectively prevented SR-Gut-aGVHD in murine models. *Ceacam1* deficiency on IECs resulted in (i) higher numbers of IL-22^+^IL-10^+^Foxp3^+^CD4^+^ peripheral Tregs (pTregs) and lower numbers of conventional IL-22^+^CD4^+^ T (Tcon), Th/Tc1, and Th17 cells in the intestine; (ii) higher prevalence of beneficial commensal bacteria that augment colonic pTreg expansion, with lower prevalence of pathogenic bacteria; and (iii) higher numbers of antiinflammatory CD103^–^CX3CR1^hi^ MNPs that produce indoleamine 2,3-dioxygenase (IDO) and IL-10, with lower numbers of proinflammatory CD103^+^CX3CR1^lo^ MNPs that produce IL-6. Thus, specifically targeting IEC CEACAM1 represents a promising approach for prevention of SR-Gut-aGVHD.

## Abstract 2

Targeting CEACAM1 on intestinal epithelial cells prevents steroid-refractory gut acute graft-versus-host disease via the expansion of pTreg cells and anti-inflammatory CX3CR1^hi^ MNP.

## Graphical abstract

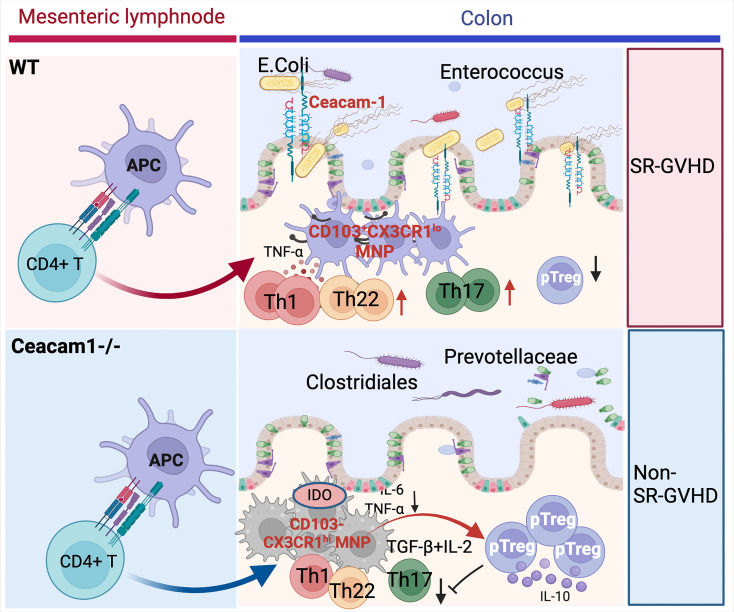

## Introduction

The gastrointestinal (GI) tract has been found to be involved in acute graft-versus-host disease (aGVHD) in up to 60% of cases (1–3). The first-line therapy for aGVHD is systemic glucocorticoid–based (GC-based) immunosuppressive regimens (4–6); however, it is only effective in approximately 50% of cases, and a significant proportion of patients with aGVHD exhibit suboptimal or absent responses (7, 8). The absence of clinical improvement after 4 weeks of high-dose GC therapy (typically prednisone 1.0–2.0 mg/kg/d) is defined as steroid-refractory gut aGVHD (SR-Gut-aGVHD) (4, 9), which develops in approximately 15% of patients with aGVHD (9, 10) and carries a dismal prognosis, with mortality exceeding 75% (4). Current effective therapeutic options for SR-Gut-aGVHD are severely limited (8), with ruxolitinib representing the sole FDA-approved second-line agent (11). The pathogenesis of SR-Gut-aGVHD remains inadequately characterized, and the critical knowledge gap and urgent unmet clinical need necessitate a deeper understanding of the molecular mechanisms.

SR-Gut-aGVHD is fundamentally initiated by pretransplantation conditioning regimens (e.g., total body irradiation [TBI]), which inflict significant damage upon the GI tract and result in release of bacterial LPS, activating TLRs and triggering proinflammatory cytokine cascades that amplify aGVHD pathogenesis (12). Importantly, GVHD itself further degrades intestinal barrier function, creating a deleterious synergy between conditioning-induced damage and subsequent alloimmune attack on the epithelium (13). This dysregulation manifests as a characteristic loss of microbial diversity, marked by the expansion of pathogenic orders such as Enterobacteriales (e.g., *Escherichia*, *Klebsiella*) and Lactobacillales, concurrent with depletion of beneficial, obligately anaerobic Clostridiales within the Firmicutes phylum (14–16). Critically, utilizing our recently established murine model of SR-Gut-aGVHD, we identified that pathogenic expansion of donor-derived IL-22^+^ T cells, dysbiosis, concomitant bacterial translocation, and reduction in protective CX3CR1^hi^ mononuclear phagocytes (MNPs) contribute to SR-Gut-aGVHD pathogenesis (17).

CEACAM1 is a member of the carcinoembryonic antigen-related cell adhesion molecule family that mediates cell-cell interactions and cellular signaling events (18). CEACAM1 is expressed by epithelial, endothelial, lymphoid, and myeloid cells (19–21). In mice and humans, CEACAM1 on T cells is a coinhibitory receptor (19, 22–25). However, more recent reports showed that CEACAM1 has 2 isoforms, short chain (S) and long chain (L); CEACAM1-S expressed on intestinal T cells provides costimulatory signals, whereas CEACAM1-L is coinhibitory (26, 27). In the setting of GVHD, CEACAM1 deficiency in donor T cells or recipients augmented GVHD, while CEACAM1-overexpressing in donor T cells ameliorated GVHD (28). In addition, CEACAM1 expressed by IECs functions as a receptor for pathogenic bacteria and viruses (29, 30); and CEACAM1-specific adhesins allow various bacteria and viruses to attach to, invade, or transcytose through cells and to polarize epithelial and endothelial cells, thereby influencing their ability to colonize their hosts (31). These pathogens might also exploit the immunoregulatory capacity of CEACAM1 to affect the function of T, B, and dendritic cells in other ways (18). IEC expression of CEACAM1 is triggered by the inflammatory cytokines IFN-γ, TNF-α and IL-1β, as well as adherent-invasive *E*. *coli* (AIEC) (32, 33).

The role of intestinal epithelial CEACAM1 in the setting of aGVHD, especially in SR-Gut-aGVHD, has not been previously explored. Since dysbiosis and bacteria translocation play an important role in the pathogenesis of SR-Gut-aGVHD, we investigated the role of intestinal epithelial CEACAM1 in regulating the microbiome profile as well as Th and CX3CR1^+^ MNP differentiation. We observed high expression of intestinal epithelial CEACAM1 in SR-Gut-aGVHD in patients and in murine models. Deletion of *Ceacam1* on host intestinal epithelial cells (IECs) blocked pathogen penetration, increased levels of Treg-inducing beneficial bacteria, expanded IDO^+^IL-10^+^CD103^–^CX3CR1^hi^ regulatory MNPs but reduced IL-6^+^CD103^+^CX3CR1^lo^ proinflammatory MNPs, leading to expansion of peripheral Tregs (pTregs) and prevention of SR-Gut-aGVHD.

## Results

### Colonic epithelial expression of CEACAM1 and infiltration of IL-22^+^ T cells are enhanced in patients with SR-Gut-GVHD.

**Figure 1:**
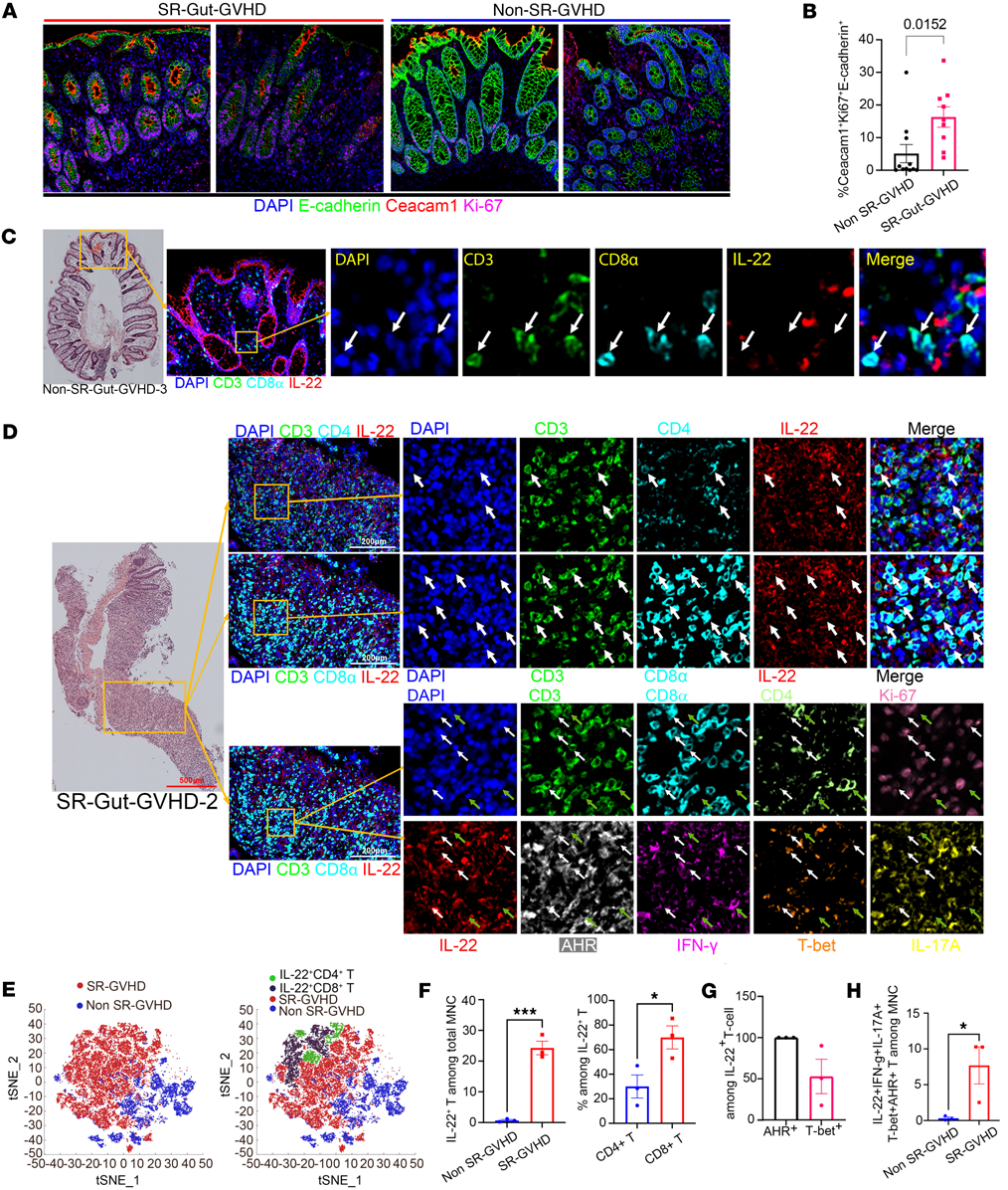
Colonic epithelial expression of CEACAM1 and infiltration of IL-22^+^IFN-γ^+^ T cells are enhanced in patients with SR-Gut-GVHD. Using H&E staining and image mass cytometry (IMC), we evaluated the severity of tissue damage indicated by immune cell infiltration and CEACAM1 expression on epithelial cells in colon biopsies from SR-Gut-GVHD (*n* = 3) and Non–SR-GVHD patients (*n* = 3). The IMC scan was based on 1 µm per pixel. The H&E overview was acquired withZEISS ZEN Tile Scan, and the magnification of the indicated regions are shown with yellow boxes (scale bars) on the right. (**A**) Representative IMC staining patterns of CEACAM1, Ki-67, and E-cadherin in patient colon biopsy samples. Scale bar: 200 μm. (**B**) Percentage of CEACAM1^+^Ki-67^+^E-cadherin^+^ cells among total MNCs. The *P* value is shown. (**C**) Representative IMC staining pattern of colon biopsy from a non-SR-Gut-GVHD patient. The panel shows staining for DAPI (blue), CD3 (green), CD8α (cyan), and IL-22 (red). White arrows indicate representative CD8^+^ T cells. (**D**) Representative IMC staining patterns for analysis of IL-22^+^CD4^+^ and IL-22^+^CD8^+^T cells in a colon biopsy sample from a patient with SR-Gut-GVHD. The arrows in the upper rows of the two arrays of panels indicate representative IL-22^+^CD4^+^ or IL-22^+^CD8^+^ T cells. The lower rows provide a more detailed analysis of T cell subsets from the same patient. The white arrows indicate IL-22^+^CD8^+^ T cells with expression of Ahr, IFN-γ, T-bet, or IL-17A, while the green arrows indicate CD4^+^ T cells with or without expression of Ahr, IFN-γ, T-bet, or IL-17A. **(32. AUTHOR: Please edit to specify the total magnifications for the images in C and D.)** (**E**) General cellular distribution in the t-distributed stochastic neighbor embedding (t-SNE) map of all MNCs (left panel) and IL-22^+^CD4^+^ and IL-22^+^CD8^+^ T cells (right panel). (**F**) Percentages of IL-22^+^ T cells among total MNCs (left panel), CD4^+^ and CD8^+^ T among IL-22^+^ T cells in SR-Gut-GVHD, and non-SR-Gut-GVHD tissues. (**G**) Percentages of Ahr^+^ and T-bet^+^ cells among IL-22^+^ T cells. (**H**) Percentages of IFN-γ^+^IL-17A^+^Ahr^+^T-bet^+^IL-22^+^ T cells among total MNCs. *P* values were calculated by unpaired 2-tailed Student’s *t* tests (**B**, **F**-**H**). *P < 0.05; ***P < 0.001.

We measured CEACAM1 expression on IECs and T cell cytokine profiles of colon biopsy samples from patients with SR-Gut-GVHD as compared with steroid-responsive Gut-GVHD samples. We stained biopsy sections from patients with or without SR-Gut-GVHD with H&E to assess the severity of tissue damage (Supplemental Figure 1A; supplemental material available online with this article; https://doi.org/10.1172/jci.insight.186984DS1). Colonic epithelial cells were identified by E-cadherin expression, and cell proliferation was identified by Ki-67 expression. Numbers of Ki-67^+^CEACAM1^+^E-cadherin^+^ cells in the colon tissues were higher in SR-Gut-GVHD than non-SR-GVHD samples (Figure 1, A and B).

We assessed the spatial distribution of colon tissue-infiltrating immune cells, including CD4^+^ T, CD8^+^ T, and myeloid cells in situ, with imaging mass cytometry (IMC) (34, 35). A panel of 18 antibodies (Supplemental Table 1) combined with collagen type I staining was used to visualize the extracellular matrix of the basement membrane. The epithelium and lamina propria were distinguished as pan-keratin^+^E-cadherin^+^ and pan-keratin^–^E-cadherin^–^, respectively (Supplemental Figure 1, B and C). SR-Gut-GVHD colon tissues showed severe intestinal epithelial damage, as indicated by the paucity of pan-keratin^+^E-cadherin^+^ cells, while many pan-keratin^+^E-cadherin^+^ cells were found in non-SR-GVHD samples (Supplemental Figure 1, B and C). CD4^+^ and CD8^+^ T cell infiltration was much more prominent in SR-Gut-GVHD as compared with non-SR-GVHD; most CD4^+^ and CD8^+^ T cells colocalized with CD11b^+^ myeloid cells in the lamina propria (Supplemental Figure 1, B–D). Differential expression of CD45RA and CD45RO allowed for further discrimination of CD45RA^+^CD45RO^–^ naive T (T_N_) cells from CD45RA^–^CD45RO^+^ memory T (Tm) cells in lamina propria. Most of the CD3^+^CD4^+^ or CD8^+^ T cells were Tm cells regardless of GVHD severity (Supplemental Figure 1, B and C). In SR-Gut-GVHD, most of the T cells were Ki-67^+^ and granzyme B^+^, indicating their proliferation capacity and cytotoxicity function. In contrast, non-SR-GVHD colon tissues contained only small numbers of Ki-67^+^ and granzyme B^+^ T cells (Supplemental Figure 1, B and C).

Consistent with our previous studies (17), the percentages of IL-22^+^CD4^+^ or CD8^+^ T cells were higher in SR-Gut-aGVHD as compared with non-SR-GVHD colon sections, and there were more CD8^+^ than CD4^+^ among the IL-22^+^ T cells (Figure 1, C–F). All IL-22^+^CD8^+^ T cells in the SR-Gut-aGVHD expressed Ahr but some of them variably coexpressed T-bet, IFN-γ, or IL-17A (Figure 1, D, G, and H). Taking together, these observations suggest that infiltrating T cells in the colon tissues from patients with SR-Gut-GVHD had increased Th/Tc22-like cells in association with increased expression of Ceacam1 on colonic epithelial cells.

### Host Ceacam1 deficiency ameliorates SR-Gut-aGVHD but does not affect GVHD in the absence of steroid treatment.

Arnhold et al. recently reported impaired epithelial regeneration in immune-mediated intestinal damage in recipient mice given daily peritoneal injection of 1–6 mg/kg prednisolone from days 1 to 28 after HCT (36). A question was raised whether 4 i.v. injections of 5 mg/kg dexamethasone (DEX) (4-DEX) impaired IEC regeneration in the absence of GVHD in our SR-Gut-GVHD model (17). Thus, we compared the 4-DEX treatment on BALB/c recipients given syngeneic BALB/c or allogeneic C57BL/6 grafts. We observed that the 4-DEX treatment did not cause obvious damage in the small intestine or colon epithelial cells of recipients given a syngeneic graft, although it caused severe colon tissue damage in the recipients given an allogeneic graft (Supplemental Figure 2). This is consistent with our previous report (17) and the recent report that prolonged steroid treatment augmented alloimmune-mediated impairment of intestinal epithelial regeneration (36).

**Figure 2:**
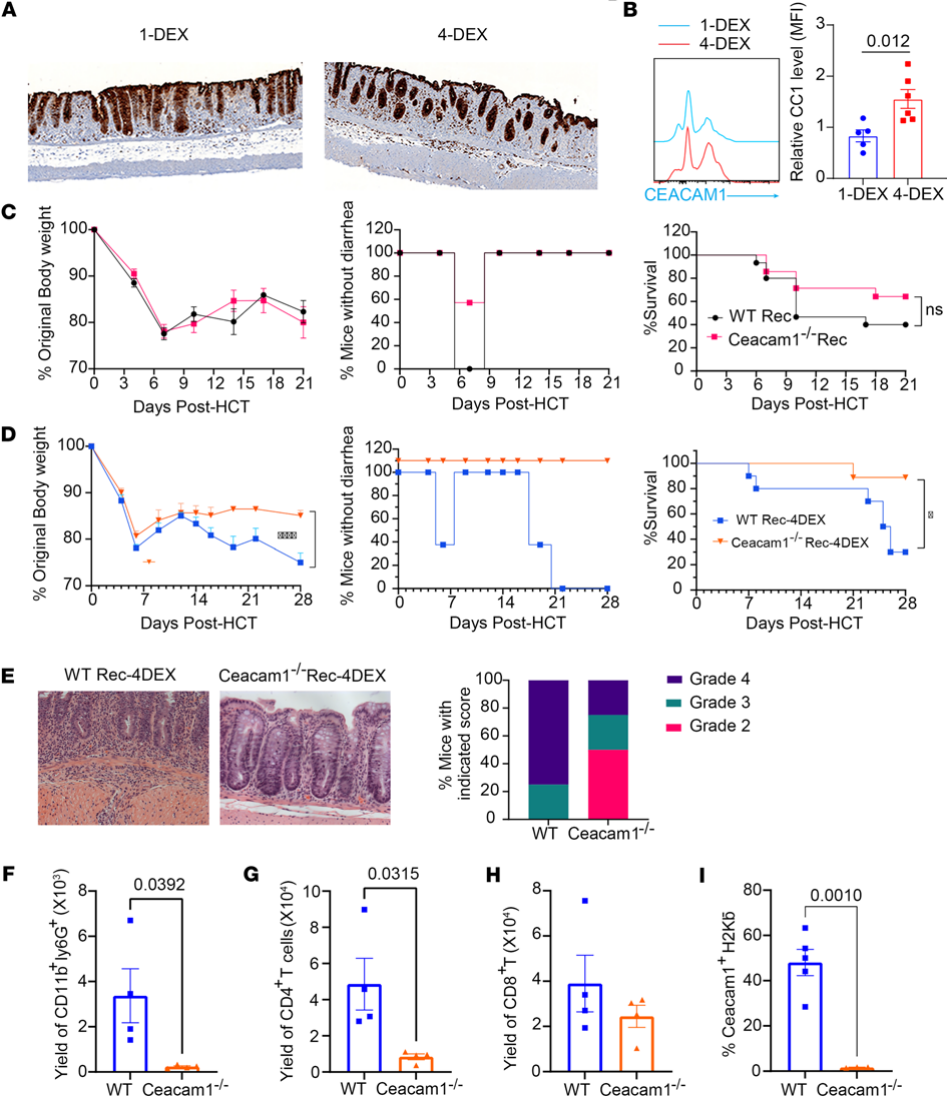
Host *Ceacam1* deficiency ameliorates SR-Gut-aGVHD but not untreated GVHD. (**A** and **B**) BALB/c recipients received T cell–depleted BM (TCD-BM) with or without splenocytes from WT C57BL/6 donors. Recipients of TCD-BM and splenocytes received 1 or 4 DEX injections after HCT. Colon epithelial CEACAM1 expression was analyzed on day 25 by IHC and flow cytometry. (**A**) Representative images. One representative micrographic photo (original magnification, ×100) is shown of 4 replicate mice in each group. (**B**) MFI of CEACAM1. *n* = 5–6 combined from 2 replicate experiments. (**C**) WT (WT Rec) or *Ceacam1^–/–^* BALB/c (*Ceacam1^–/–^* Rec) recipients received WT C57BL/6 splenocytes and TCD-BM (untreated GVHD). Original body weight, mice without diarrhea, and survival are shown as percentages. *n* = 15 from 2 replicate experiments. (**D**) Experimental conditions as in **C**, except that recipients received 4-DEX injection (SR-Gut-aGVHD). *n* = 10 from 2 replicate experiments. (**E**) Colon histopathology was evaluated on day 25. Representative micrographic photos (original magnification, ×200) and percentage of mice with indicated histopathological scores. *n* = 4/group from 2 replicate experiments. (**F**) Mean ± SEM of yield of CD11b^+^Ly6G^+^ cells. (**G**) Means ± SEM of yield of H2Kb^+^TCRβ^+^CD4^+^ T cell. *n* = 4 mice/group from 2 experiments. (**H**) Means ± SEM of %CEACAM1^+^H2Kb^–^ cells. (**I**) IHC staining of CEACAM1 (purple), CD11b (yellow) and CD3 (teal) in colon on day 25. Representative photomicrographs (original magnification, x100) are shown. The *P* value is shown in **B**, **F**, **G**, and **I**. Nonlinear regression (curve fit) was used for body weight comparisons. Log-rank test was used for survival comparisons. **B** and **F**–**I**: unpaired 2-tailed Student’s *t* test).

Next, since we observed high expression of Ceacam1 by colon epithelial cells of patients with SR-gut-GVHD (Figure 1A), we tested whether this was the case in SR-Gut-GVHD mice. As compared with 1-DEX recipients, the intensity of CEACAM1 staining appeared higher in the colonic epithelium of SR-Gut-aGVHD recipients (4-DEX), and the MFI levels of CEACAM1 were also much higher than in 1-DEX recipients (Figure 2, A and B). These results suggest that consistent with SR-Gut-GVHD in patients, SR-Gut-GVHD in murine models was associated with enhanced expression of CEACAM1 by colonic epithelial cells.

In MHC-mismatched murine models, the absence of CEACAM1 on donor T cells exacerbates GVHD, with higher alloreactive T cell infiltration in the gut (28); loss of Ceacam1 in the host also leads to increased sensitivity to radiation damage and increased infiltration of donor T cells in the lower GI tract of GVHD recipients (28). Consistent with these results, we found no reduction of severity of GVHD in *Ceacam1^–/–^* compared with WT recipients, as suggested by the observation that there was little difference in body weight loss or diarrhea; and in both groups, approximately 40%–60% of the recipients died within 3 weeks after HCT (Figure 2C). These results indicate that *Ceacam1* deficiency in recipients may augment acute gut GVHD in the absence of steroid treatment.

Since host colonic epithelial cell expression of CEACAM1 was higher in recipients with SR-Gut-aGVHD (Figure 2, A and B), we tested the role of host CEACAM1 in induction of SR-Gut-aGVHD. WT or *Ceacam1^–/–^* BALB/c mice were given transplants from C57BL/6 donors, and the recipients received 4-DEX treatment. While all WT recipients showed diarrhea, none of the *Ceacam1^–/–^* recipients showed diarrhea, and they had higher body weight and higher survival rate (90% versus 30%) by day 28 after HCT (Figure 2D); as well as reduced colonic inflammation and epithelial damage, with better preservation of intestinal crypt structure and lower numbers of infiltrating donor CD11b^+^Ly6G^+^ neutrophils and CD4^+^ but not CD8^+^ T cells in the colon tissues as compared with WT recipients (Figure 2, E–H). The lack of CEACAM1 expression on the colonic epithelial cells of *Ceacam1^–/–^* recipients was validated by flow cytometry and IHC staining (Figure 2I). These results indicate that host but not donor cell CAECAM1 plays an important role in regulating SR-Gut-aGVHD and that *Ceacam1* deficiency on host cells ameliorates SR-Gut-aGVHD in association with reduction of infiltrating *Ceacam1^+^* donor T cells and neutrophils in colon tissues.

### SR-Gut-aGVHD is ameliorated by Ceacam1 deficiency on host IECs but not on hematopoietic cells.

As CEACAM1 is expressed by both hematopoietic and nonhematopoietic cells, the type of host cells associated with SR-Gut-aGVHD was unclear. To address this question, we established bone marrow chimeras with *Ceacam1* deficiency only on parenchyma cells, including IECs (IEC-*Ceacam1^–/–^* chimeras) and hematopoietic cells (HC-*Ceacam1^–/–^* chimeras); WT chimeras were established as control. WT chimeras, HC-*Ceacam1^–/–^* chimeras, and IEC-*Ceacam1^–/–^* chimeras were used to test the development of SR-Gut-aGVHD, as previously described (17).

**Figure 3:**
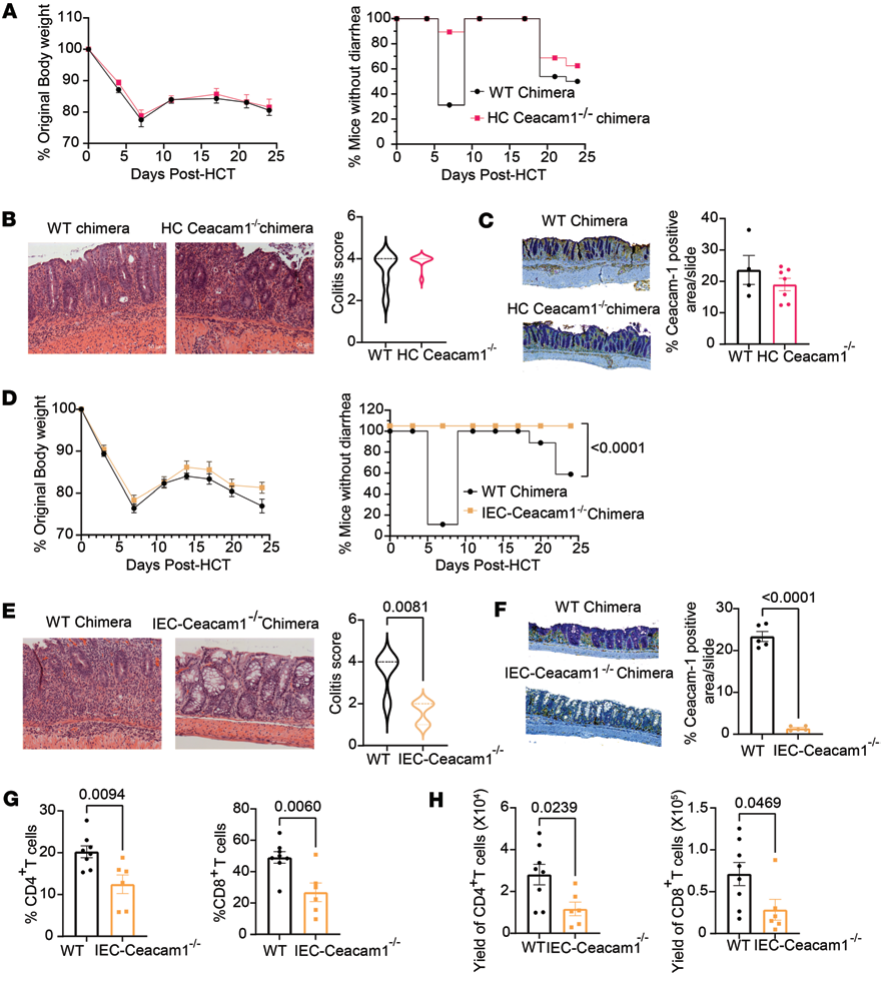
SR-Gut-aGVHD is ameliorated by *Ceacam1* deficiency on host IECs but not on hematopoietic cells. (**A**–**C**) WT BALB/c recipients received TCD-BM from WT or *Ceacam1^–/–^* BALB/c donors to generate the chimeras. Two months after bone marrow reconstitution, WT or HC-*Ceacam1^–/–^* chimeras were lethally irradiated and engrafted with splenocytes and TCD-BM from WT C57BL/6 donors, and the recipients were given 4-DEX treatment. (**A**) Original body weight and mice without diarrhea are shown as percentages. *n* = 18 (WT chimeras), 15 (host HC-*Ceacam1^–/–^* chimeras) from 2 replicate experiments. (**B**) Histopathology of colon on day 25. Representative photomicrographs (original magnification, ×200) and percentage of mice with indicated histopathological scores. *n* = 6 (WT), *n* = 8 (HC-*Ceacam1^–/–^*), from 2 replicate experiments. (**C**) IHC staining of CEACAM1 (purple), CD11b (yellow) and CD3 (teal) on colon day 25 after HCT. Representative photomicrographs (original magnification, ×100). Mean ± SEM percentage of CEACAM1^+^ area relative to the whole slide. (**D**–**H**) WT and *Ceacam1^–/–^* BALB/c recipients received TCD-BM from WT BALB/c donors to generate chimeras. Then the irradiated WT or IEC-*Ceacam1^–/–^* chimeras received splenocytes and TCD-BM from WT C57BL/6 donors. (**D**) Original body weight and mice without diarrhea are shown as percentages. *n* = 18 (WT), 15 (IEC-*Ceacam1^–/–^*), from 2 replicate experiments. (**E**) Histopathology of colon on day 25. Representative photomicrographs (original magnification, ×200) and percentage of mice with indicated histopathological scores. *n* = 4 (WT), 5 (IEC-*Ceacam1^–/–^*), from 2 replicate experiments. (**F**) IHC staining of CEACAM1 (purple), CD11b (yellow), and CD3 (teal) on colon on day 25. Representative photomicrographs (original magnification, ×100). Mean ± SEM percentage of CEACAM1^+^ area relative to the whole slides. (**G** and **H**) Mean ± SEM percentage (**G**) and yield of CD4^+^ and CD8^+^ T cells (**H**) in colon intraepithelial tissue. *P* values are shown in **E**–**H**. **D**: nonlinear regression, curve fit; **E**–**H**; unpaired 2-tailed Student’s *t* test.

By comparing HC-*Ceacam1^–/–^* chimeras with WT chimeras, we observed no differences in body weight loss, diarrhea, colonic histopathology, or colonic expression of CEACAM1 as measured with IHC (Figure 3, A–C). However, by comparing IEC-*Ceacam1^–/–^* chimeras with WT chimeras, we observed that nearly all WT chimeras but no *Ceacam1^–/–^* chimeras developed diarrhea, although there were no differences in body weight loss (Figure 3D). On day 25 after HCT, the IEC-*Ceacam1^–/–^* chimeras showed much less inflammation and damage to crypts and glands as compared with WT chimeras (Figure 3E). The lack of CEACAM1 expression on host colonic epithelial cells in IEC-*Ceacam1^–/–^* chimeras was validated by IHC staining (Figure 3F). The percentage and yield of both donor-type CD4^+^ and CD8^+^ T cells were significantly lower in colon tissue of IEC-*Ceacam1^–/–^* chimeras than in that of WT chimeras (Figure 3, G and H). These results indicate that *Ceacam1* deficiency in host intestinal epithelial but not hematopoietic cells prevents the induction of SR-Gut-aGVHD.

### Amelioration of SR-Gut-aGVHD by Ceacam1 deficiency in IECs is associated with distinct CD4^+^ Tcon cell and pTreg subsets and gene signaling pathways in mesenteric lymph node cells.

**Figure 4:**
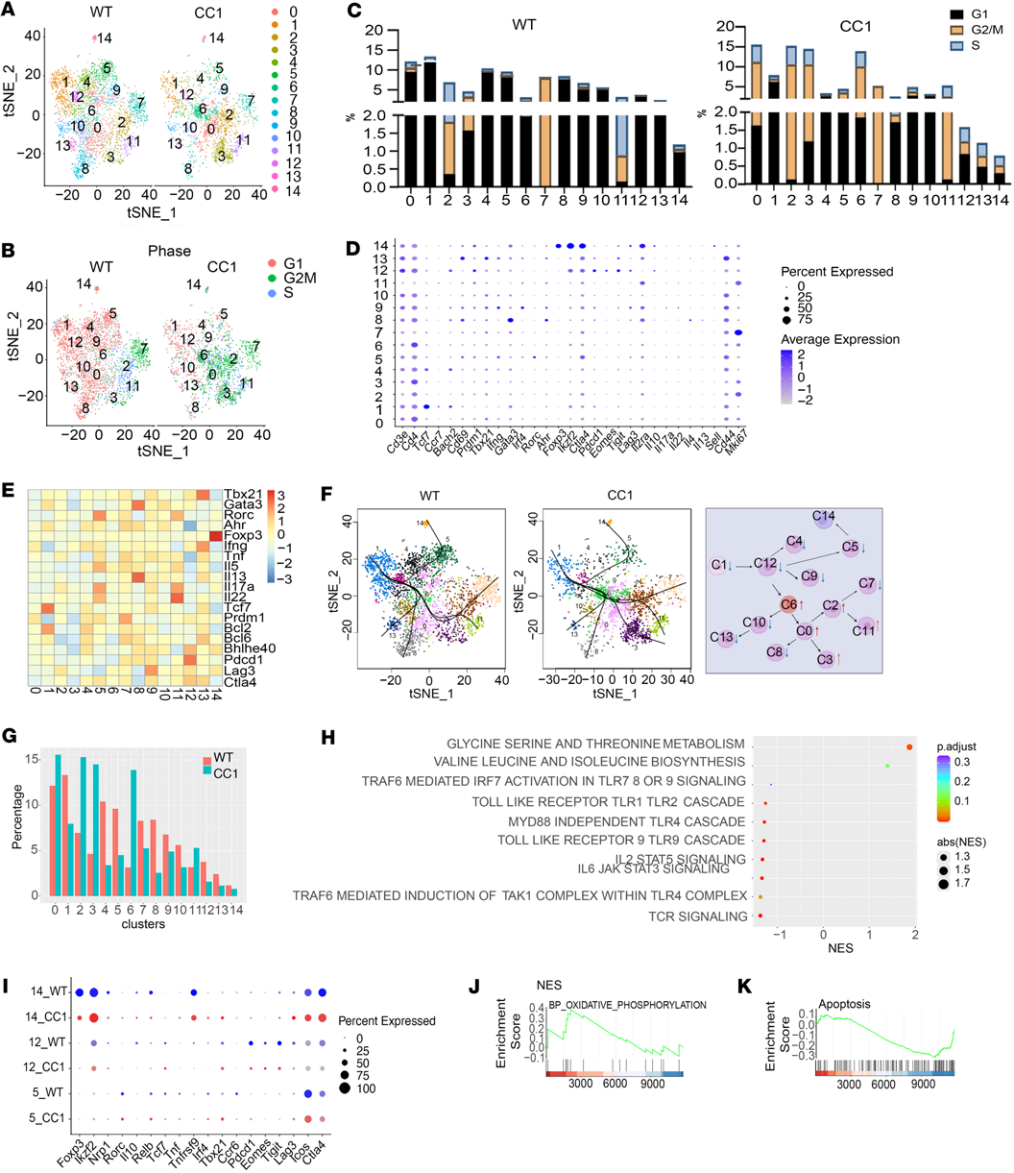
Amelioration of SR-Gut-aGVHD by *Ceacam1* deficiency in IECs is associated with distinct CD4^+^ Tcon cells and pTreg subsets and gene signaling pathways in MLN cells. WT and IEC-*Ceacam1^–/–^* (CC1*^–/–^*) chimeras were engrafted with donor cells and treated with 4-DEX as described for Figure 3. On day 25, CD4^+^ T cells from MLNs of WT or IEC-CC1*^–/–^* recipients were sorted for scRNA plus ATAC sequencing. (**A**) Subclustering t-SNE of all CD4^+^ T cells. The 15 clusters are indicated. (**B**) t-SNE plot of cell cycle. (**C**) Comparison of percentage of individual cluster of cell cycle. (**D**) Dot plot of immunomodulatory genes across all clusters. (**E**) Heatmap showing the differential transcription factor motif accessibility. (**F**) t-SNE showing the lineage trajectory ordered from naive cells to Tregs (cluster 14) or effector T cells (other clusters). The diagram shows changes in CD4^+^ T clusters in *Ceacam1^–/–^* chimeras compared with WT chimeras. ↑, increase; ↓, decrease; C1, naive T cells; C3, cycling CD4^+^ T cells (*Ceacam1^–/–^* chimera only); C5, G_1_ resting Th17; C7, Th1-, Th2-, Th17-, and Th22-like; C8, Th2; C11, G_2_M/S proliferating Th17; C12, anergic Th1; C13, Th1; C14, pTregs. (**G**) Comparison of percentages of individual clusters. (**H**) Bubble plot showing enriched KEGG, REACTOME, and HALLMARK signature pathways by comparing IEC-CC1*^–/–^* and WT. (**I**) Dot plot of immunomodulatory genes of clusters 5, 12, and 14, comparing IEC-CC1*^–/–^* and WT. (**J** and **K**) GSEA plot of HALLMARK apoptotic pathway (**J**) and BP_GO oxidative phosphorylation pathway (**K**) of cluster 14 by comparing IEC-CC1*^–/–^* recipients with WT recipients. abs(NES), absolute value of normalized enrichment score.

To investigate the mechanisms whereby *Ceacam1* deficiency on IECs prevented induction of SR-Gut-aGVHD, we analyzed sorted donor-type CD4^+^ but not CD8^+^ T cells from the MLN of WT or IEC-*Ceacam1^–/–^* chimeras with scRNA-Seq combined with assay for transposase-accessible chromatin sequencing (ATAC-Seq). scRNA-Seq analysis identified 15 clusters (i.e., 0–14) (Figure 4A). In WT chimeras, most clusters (i.e., 0,1, 4–6, 8–10,12–14) were G_1_-phase dominant; clusters 2, 7, and 11 were G_2_/M-S–phase dominant; and cluster 3 had mixed phases (Figure 4, B and C). IEC-*Ceacam1^–/–^* chimeras showed altered cycling: clusters 1, 4, 5, and 8–10 remained G_1_ dominant, but clusters 0, 3, 6, and 12–14 shifted to mixed-phase (vs. G_1_-dominant in WT). Consequently, levels of G_2_/M-S–phase cells significantly increased in clusters 0, 2, 3, 6, and 12–14 (Figure 4, B and C).

With analysis of gene expression and chromatin accessibility of stemness/activation markers (Tcf7, CCR7, CD69, IL-2Rα) and Th lineage factors (Tbx21, Gata3, Rorc, Ahr, Foxp3, Ifng, Il17a, Il22, Il4, Il13) (Figure 4, D and E, and Supplemental Figure 3, A–C), we defined the clusters (C) as follows: C1 (naive T) had high Tcf7/CCR7/CD62L but low CD44/CD69/IL-2Rα expression; C0, -2, -6, -9, and -10 (activated intermediate-stage T) had downregulated naive markers, elevated CD44/Ki-67, and reduced inflammatory cytokines (Ifng and Tnf); C5 (resting Th17) and C11 (proliferating Th17) had high Rorc/IL17A/IL22; C7 (Th1/Th2/Th17/Th22-like) exhibited intermediate T-bet/Gata3/Rorc/Ahr/cytokines; C8 (Th2) had elevated Gata3/IL13; C13 (Th1) expressed high T-bet/IFN-γ; and C14 (Tregs) showed Foxp3/IL2Rα/Ctla4/IL10.

To infer CD4^+^ T ontogeny, we used the Slingshot algorithm to order single cells in all clusters in pseudotime (Figure 4F). This analysis revealed distinct trajectories of CD4^+^ T differentiation paths in WT chimeras and IEL-*Ceacam1^–/–^* (IEL-CC1*^–/–^*) chimeras. As described in the pseudopath and diagram in Figure 4F, C1 naive CD4^+^ T cells differentiate through intermediate stages (C12, -6, and -10) to C13 T-bet^+^IFN-γ^+^ Th1 cells or C8 Gata3^+^IL-13^+^ Th2 cells; through intermediate stages (C12, -6, -0, and -2) to C7 Th1/Th2/Th17/Th22-like cells or C11 G_2_M/S proliferating Rorc^+^IL-17A^+^IL-22^+^ Th17 cells; through intermediate stage (C12, -6, and -0) to C3 cycling Th subset. Alternatively, C1 naive CD4^+^ cells differentiate through C12 anergic/exhausted Th1 cells and G_1_ resting C5 IL-22^+^IL-17^+^ Th17 cells to C14 FoxP3^+^ Tregs, suggesting that these Tregs represent pTregs, as previously described by others (37, 38). Compared with WT chimeras, IEL-CC1*^–/–^* chimeras exhibited a reduction in frequencies of C1 naive, C13 terminally differentiated Th1, C8 Th2, C5 Th17, and C7 Th1/2/17/22-like, and C12 anergic/exhausted Th1 cells; increase in C0, -2, and -6 intermediate-stage T, C3 cycling Th, and C11 proliferating Th17 cells; and little difference in C14 Tregs (Figure 4, F and G). These suggest that the absence of SR-Gut-GVHD in IEL-CC1*^–/–^* chimeras could be attributed to altered T cell differentiation and expansion.

Next, we explored the signaling and metabolic pathways that could account for the pattern of expansion and reduction of different CD4^+^ T clusters in IEL-CC1*^–/–^* chimeras. Intestinal epithelial CEACAM1 mediates bacterial translocation into intestinal tissues (29, 30), and bacterial antigens and metabolites regulate T cell differentiation and expansion (39–41). TLR signaling augments T cell differentiation into proinflammatory Th1, Th2, and Th17 (42, 43). Metabolites from serine augment effector T cell expansion (44), while metabolites from arginine and leucine augment effector T cell differentiation into pTregs (45). Gene pathway analysis showed that, compared with WT chimeras, CD4^+^ T subsets in C0, 2, 3, 6, and 11 of IEL-CC1*^–/–^* chimeras showed decreased expression of genes in TCR, IL-2/STAT5, IL-6/STAT3, MyD88/TLR4, TLR9, and TLR1/2 pathways. In contrast, signaling was enhanced in glycine-serine/Threonine and valine-leucine/isoleucine pathways (Figure 4H). These results suggest that the lower frequencies of terminally differentiated proinflammatory Th1, Th2, Th17, and Th22 cells in IEL-CC1*^–/–^* chimeras may be caused by lower bacterial translocation and TLR signaling, while higher frequencies of intermediate effector cells may be caused by higher metabolic activity in serine or leucine pathways.

We further compared the expression of nuclear transcription factors by anergic/exhausted C12 Th1 cells, C5 Th17 cells, and C14 Tregs from WT and IEL-CC1*^–/–^* chimeras (Figure 4I), as they were along the pseudotime differentiation path (Figure 4F). C12 anergic/exhausted Th1 cells from IEL-CC1*^–/–^* chimeras and WT chimeras did not show clear differences (Figure 4I). C5 cells of IEL-CC1*^–/–^* chimeras had slightly lower frequencies of Rorc and TCF7 but slightly higher frequencies of T-bet (Tbx21), Pdcd1, and Lag3 (Figure 4I). C14 Tregs of IEL-CC1*^–/–^* chimeras appeared to have slightly lower frequencies of FoxP3 and Nrp1 expression but slightly higher frequencies of Rorc, T-bet, and Lag3 expression (Figure 4I) as well as reduced apoptosis and enhanced oxidative phosphorylation pathway activity (Figure 4, J and K). Oxidative phosphorylation is associated with enhanced Treg function (46, 47). These results suggest that *Ceacam1* deficiency in IECs may augment transdifferentiation of Th1 or Th17 into pTregs that have enhanced regulatory activity (48, 49). Taking collectively, the results indicate that *Ceacam1* deficiency in IECs led to lower terminal differentiation and survival of proinflammatory Th1, Th17, and Th22 cells, while maintaining the differentiation or transdifferentiation and survival of pTregs in the MLN of recipients.

### Amelioration of SR-Gut-aGVHD by Ceacam1 deficiency in IECs is associated with lower numbers of pathogenic IL-22^+^CD4^+^ Tcon cells but higher numbers of IL-22^+^IL-10^+^CD4^+^ pTregs in the MLN and colon tissues.

**Figure 5:**
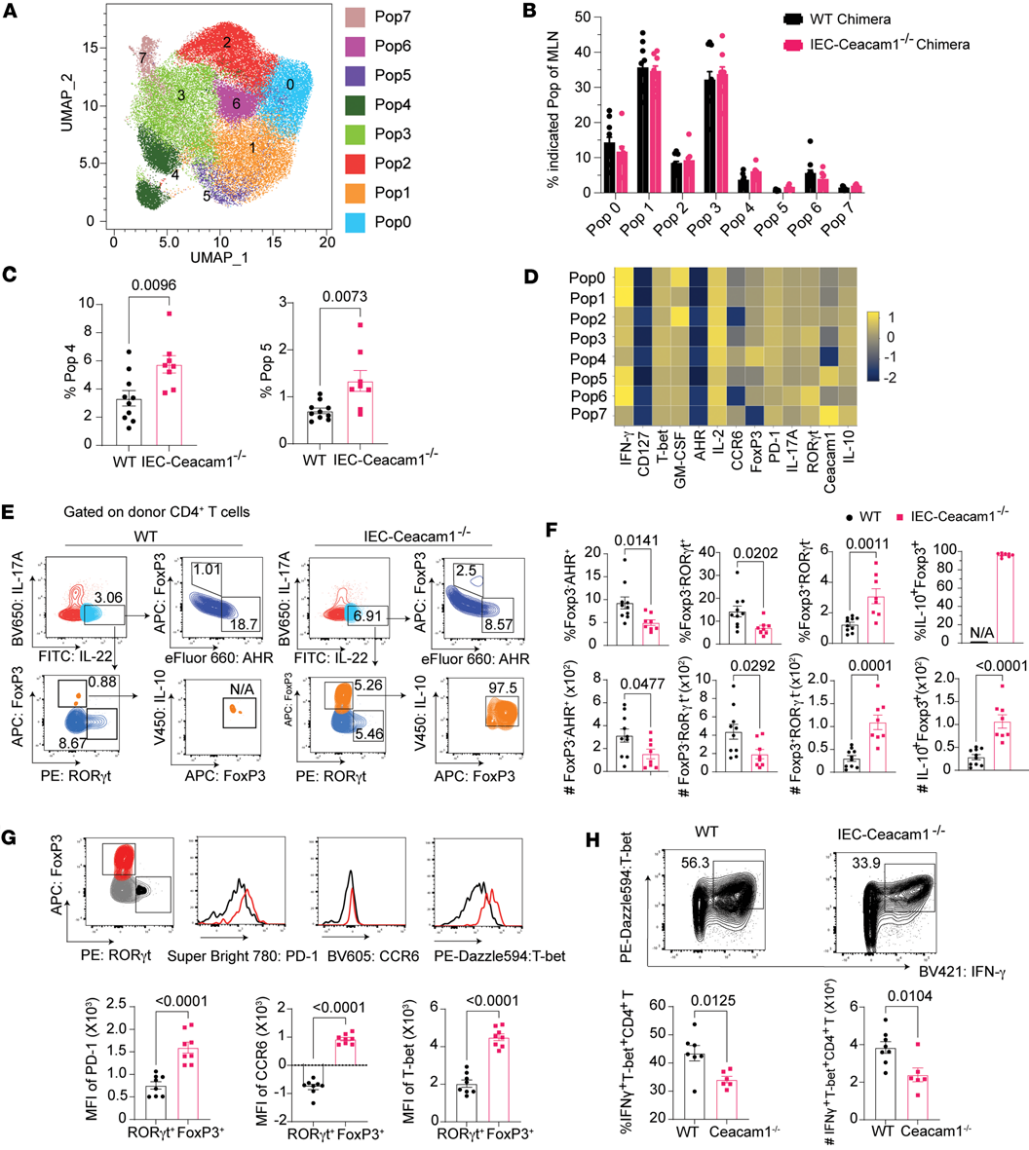
Amelioration of SR-Gut-aGVHD by *Ceacam1* deficiency in the host is associated with a reduction in IL-22^+^CD4^+^ Tcon cells but expansion of IL-22^+^IL-10^+^CD4^+^ pTregs in the MLN. WT and IEC-*Ceacam1^–/–^* chimeras were engrafted with splenocytes together with TCD-BM from WT C57BL/6 donors and induced to develop SR-Gut-GVHD, as described for Figure 2. On day 25, T cell subset in MLNs from WT or IEC-*Ceacam1^–/–^* recipients were analyzed. (**A**) UMAP plot generated from IL-22^+^IL17A^–^CD4^+^ (Th22) cells in MLNs of both WT and IEC-*Ceacam1^–/–^* chimeras. (**B**) Mean ± SEM percentages of individual populations. (**C**) Mean ± SEM percentages of populations 4 and 5 (Pop4 and Pop5). (**D**) Heatmap plot of IFN-γ, CD127, T-bet, GM-CSF, AHR, IL-2, CCR6, FoxP3, PD-1, IL-17A, RORγt, CEACAM1, and IL-10 expression. (**E**) Representative flow cytometry pattern and gating strategy of FoxP3, AHR, RORγt and IL-10 in Th22 cells. (**F**) Mean ± SEM percentage of FoxP3^–^AHR^+^, FoxP3^–^RORγt^+^, and FoxP3^+^RORγt^–^ subsets in Th22 cells and of IL10^+^ in Foxp3^+^RORγt^–^ Th22 cells; and yields of the respective subsets. (**G**) Representative flow cytometry pattern and gating strategy for PD-1, CCR6, and T-bet in FoxP3^+^RORγt^–^ and Foxp3^–^RORγt^+^ Th22 subsets. (**H**) Representative flow cytometry patterns and mean ± SEM percentage of T-bet^+^IFN-γ^+^ subset in CD4^+^ T cells and yields in WT and IEC-*Ceacam1^–/–^* chimeras. *n* = 10 (WT), *n* = 8 (IEC-*Ceacam1^–/–^*), from 2 replicate experiments. *P* values are shown in **C** and **E**–**H**. **C** and **F**–**H**: Unpaired 2-tailed Student’s *t* test.

Flow cytometry analysis on day 25 after HCT revealed comparable frequencies and yields of donor-derived Th22 (IL-22^+^IL-17A^–^CD4^+^) and Tc22 (IL-22^+^IL-17A^–^CD8^+^) cells in MLN of WT versus IEC-*Ceacam1^–/–^* chimeras (Supplemental Figure 4). High-dimensional spectral cytometry employing 21 key T cell markers (including CD127, CCR6, PD-1, CEACAM1, T-bet, RORγt, AHR, FoxP3, and cytokine markers) with uniform manifold approximation and projection (UMAP) dimensionality reduction identified 8 CD4^+^ T cell clusters demonstrating significant expansion of FoxP3^+^ cluster 4 and CEACAM1^+^ cluster 5 in IEC-*Ceacam1^–/–^* chimeras (Figure 5, A–D). Further phenotypic dissection of IL-22^+^IL-17A^–^ cells showed reduced FoxP3^–^RORγt^+^ and FoxP3^–^AHR^+^ subsets alongside expanded FoxP3^+^RORγt^–^ Tregs in IEC-*Ceacam1^–/–^* chimeras, and these Tregs exhibited near-universal IL-10 expression as well as elevated expression of PD-1, CCR6, and T-bet, which are markers conferring tissue regulatory competence (50–54). In addition, there were significantly diminished T-bet^+^IFN-γ^+^ Th1 frequencies (Figure 5, E–H). This coordinated expansion of IL-22^+^IL-10^+^FoxP3^+^ Tregs correlates mechanistically with SR-Gut-aGVHD amelioration in intestinal epithelial CEACAM1–deficient recipients.

**Figure 6:**
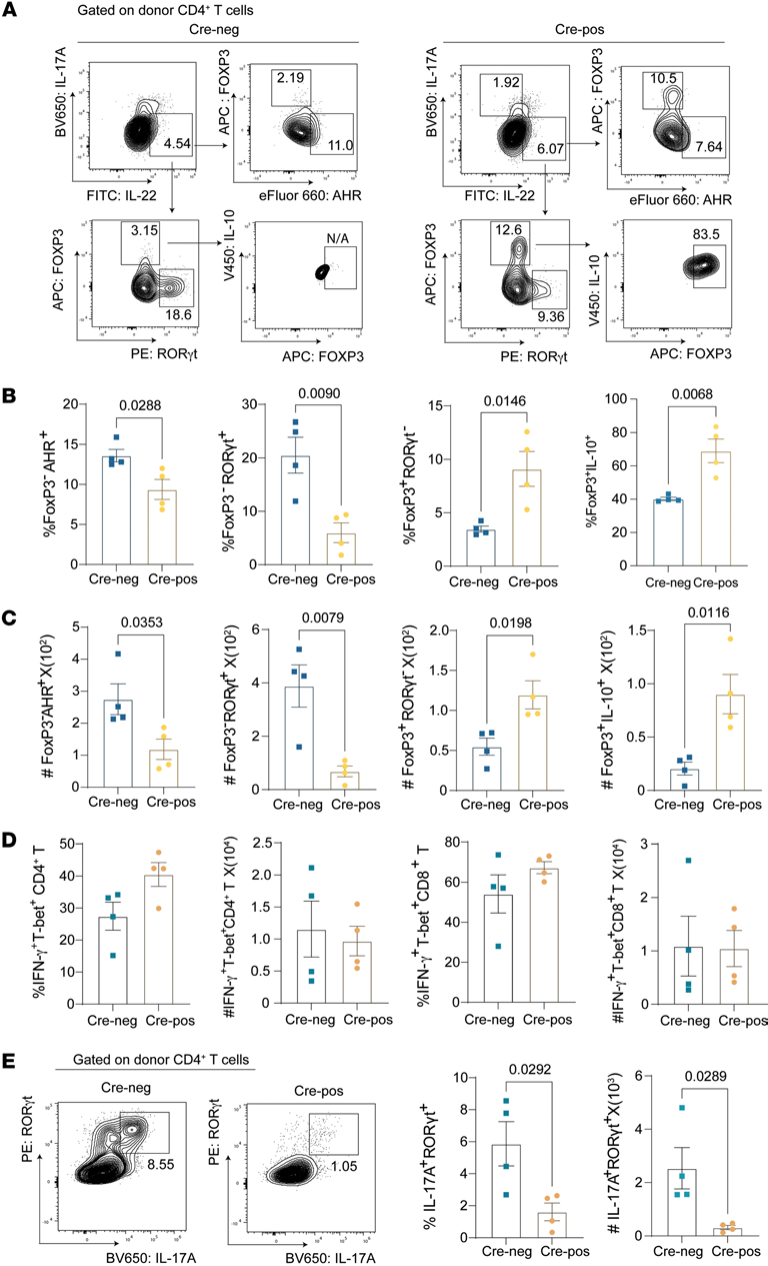
Amelioration of SR-Gut-aGVHD by *Ceacam1* deficiency specifically in host IECs validates results with WT and IEC-*Ceacam1*^–/–^ chimeras, showing lower numbers of pathogenic IL-22^+^CD4^+^ Tcon cells but higher numbers of IL-22^+^IL-10^+^CD4^+^ pTregs in the MLN. *Villin^Cre^*-positive *Ceacam1^fl/fl^* and *Villin^Cre^*-negative *Ceacam1^fl/fl^* recipients were engrafted with splenocytes together with TCD-BM from CD45.1 BALB/c donors and injected with 4-DEX. On day 21, T cell subsets in MLN from Cre-pos or Cre-neg recipients were analyzed. (**A**) Representative flow cytometry pattern and gating strategy of FoxP3, AHR, RORγt, and IL-10 expression in Th22 cells. (**B** and **C**) Mean ± SEM percentage (**B**) and yield (**C**) of FoxP3^–^AHR^+^, FoxP3^–^RORγt^+^, FoxP3^+^RORγt^–^, and IL10^+^Foxp3^+^ Th22 subsets. (**D**) Mean ± SEM percentage of T-bet^+^IFN-γ^+^ in CD4^+^ T cells and yield of T-bet^+^IFN-γ^+^ CD4^+^ T cells. (**E**) Representative flow cytometry pattern and mean ± SEM percentage of RORγt^+^IL-17A^+^ in CD4^+^ T cells and yield of RORγt^+^IL-17A^+^ CD4^+^ T cells. *n* = 4, from 2 replicate experiments. *P* values are shown in **B**, **C**, and **E**. **B**–**E**: Unpaired 2-tailed Student’s *t* test.

To validate the role of host intestinal epithelial CEACAM1 in SR-Gut-aGVHD pathogenesis, we generated *Villin^Cre^Ceacam1^fl/fl^* mice (55, 56). Compared with Cre-negative littermates, Cre-positive mice exhibited significantly attenuated GVHD severity, evidenced by improved body weight recovery and enhanced survival (Supplemental Figure 5A). Mirroring the *IEC-Ceacam1^–/–^* chimera findings (Figure 5), *Villin^Cre^Ceacam1^fl/fl^* recipients demonstrated substantially increased frequencies of IL-22^+^IL-10^+^FoxP3^+^RORγt^–^AHR^–^ CD4^+^ T cells in MLN, without significant alterations in IL-22^+^IL-17A^–^ CD4^+^ T cell populations (Figure 6, A–C, and Supplemental Figure 5B). Critically, although Th1 and Tc1 cell counts remained comparable between genotypes, we observed a dramatic reduction in IL-17A^+^ Th17 cells within *Villin^Cre^Ceacam1^fl/fl^* recipients (Figure 6, D and E). These results support the observation that Ceacam1 deficiency in IECs prevent SR-Gut-aGVHD.

### Amelioration of SR-Gut-aGVHD by intestinal epithelial Ceacam1 deficiency is associated with enrichment of IL-22^+^ pTregs among intestinal intraepithelial lymphocytes.

**Figure 7:**
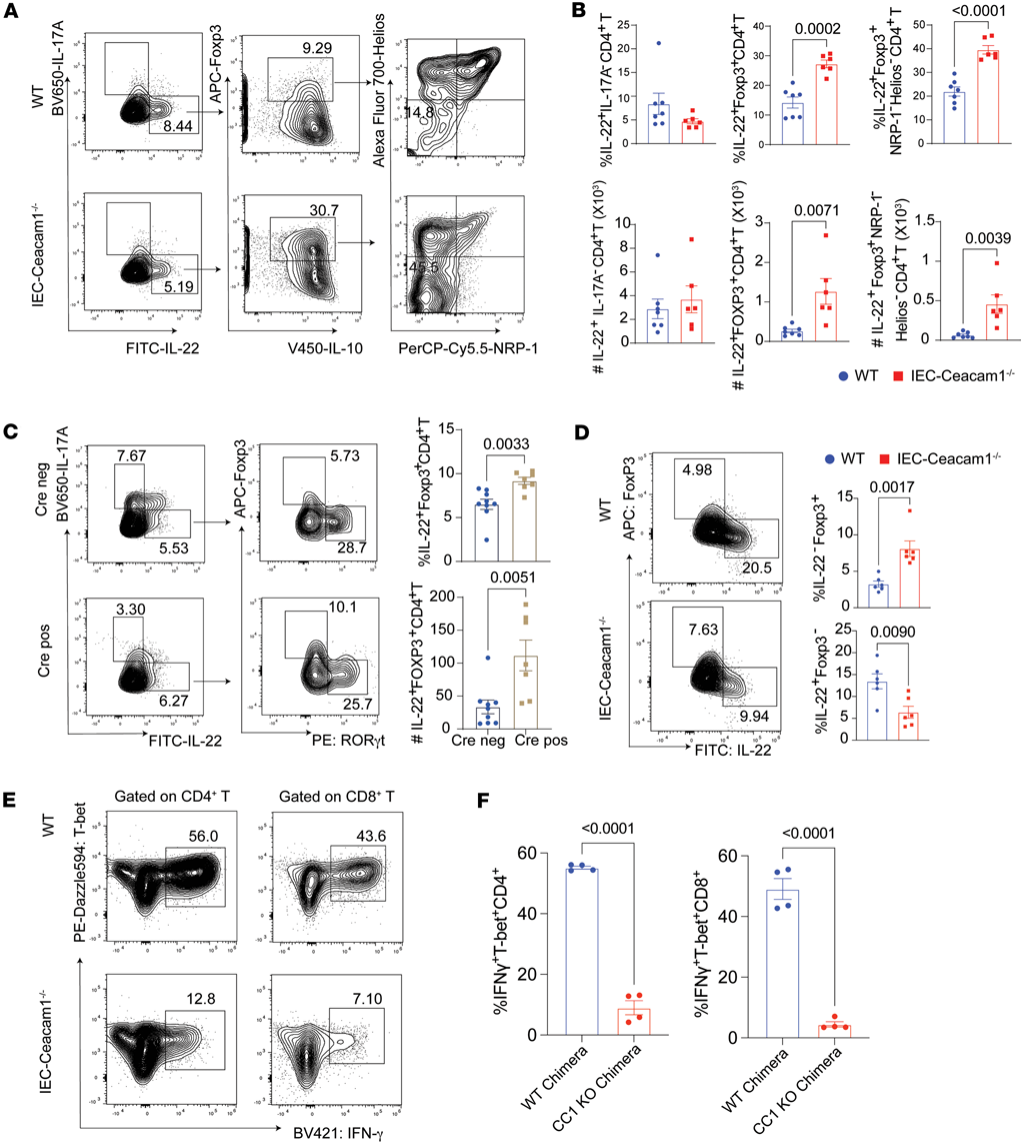
Amelioration of SR-Gut-aGVHD by intestinal epithelial *Ceacam1* deficiency is associated with enrichment of IL-22^+^ pTregs cells among intestinal intraepithelial lymphocytes. WT and IEC-*Ceacam1^–/–^* chimeras were engrafted with splenocytes together with TCD-BM from WT C57BL/6 donors as described for Figure 2. (**A**, **B**, and **D**–**F**) *Villin^Cre^*-pos-*Ceacam1^fl/fl^* and *Villin^Cre^*-neg-*Ceacam1^fl/fl^* recipients were engrafted with splenocytes together with TCD-BM from CD45.1 BALB/c donors and injected with 4-DEX. (**A**) Representative flow cytometry patterns and gating strategy of IL-22^+^Foxp3^+^IL-10^+^NRP-1^–^Helios^–^CD4^+^ T cells. (**B**) Mean ± SEM percentages and yields of %IL22^+^IL-17A^–^, IL-22^+^FoxP3^+^IL-10^+^, and IL-22^+^FoxP3^+^IL-10^+^NRP-1^–^Helios^–^ cells in the colonic epithelium. *n* = 7 (WT), 6 (IEC-*Ceacam1^–/–^*). (**C**) Representative flow cytometry patterns and mean ± SEM percentages and yields of FoxP3^+^IL-22^+^IL-17A^–^CD4^+^ T cells in colon intraepithelial tissue. *n* = 7–9. (**D**) Representative flow cytometry patterns; mean ± SEM percentages and yields of FoxP3^+^IL-22^–^ and FoxP3^–^IL-22^+^ cells in the colonic lamina propria. *n* = 4. (**E** and **F**) Representative flow cytometry patterns;mean ± SEM percentages and yields of T-bet^+^IFN-γ^+^ cells among CD4^+^ and CD8^+^ T cells in the colonic lamina propria. *n* = 4. Combined from 2 replicate experiments. *P* values are shown in **B**–**D** and **F**. Unpaired 2-tailed Student’s *t* test was used to compare 2 means.

Given the observed expansion of IL-22^+^FoxP3^+^ pTregs in MLN of intestinal epithelial CEACAM1–deficient recipients (Figures 5 and 6), we analyzed T cells in the primary SR-Gut-aGVHD target colon tissue. On day 25 after HCT, IEC-*Ceacam1^–/–^* chimeras exhibited unchanged total Th22 frequencies but significantly increased proportions and yields of FoxP3^+^IL-10^+^ Th22 subsets within the colonic epithelium. These cells displayed an NRP-1^–^Helios^–^ pTreg phenotype (Figure 7, A and B) (57, 58). Notably, *Villin^Cre^Ceacam1^fl/fl^* mice recapitulated this pattern with elevated IL-22^+^FoxP3^+^CD4^+^ T cells versus controls (Figure 7C), which validated that epithelial CEACAM1 deficiency promoted colonic pTreg accumulation among IL-22^+^CD4^+^ T cells, correlating with SR-Gut-aGVHD amelioration.

Treg-mediated suppression of GVHD is associated with IL-10 (59), and IL-10 can also be produced by Th1, Th17, or Tr1 cells (36, 60–62). The percentages and yield of IL-10^+^RORγt^–^ CD4^+^ T cells were higher in IEC-*Ceacam1^–/–^* than in WT chimeras; there were IL-10^+^IFN-γ^+^ Tr1 cells among the IL-10^+^RORγt^–^CD4^+^ T subset; but the percentage in IEC-*Ceacam1^–/–^* chimeras was lower than in WT chimeras, although the yield was not different (Supplemental Figure 6, A and B). The signature of IL-10^+^RORγt^–^ CD4^+^ T cells is consistent with the pTregs in MLN, as PD-1 and T-bet expression was upregulated while IL-17A expression was downregulated when compared with IL-10^–^RORγt^+^CD4^+^ T cells (Supplemental Figure 6C). In agreement with the observation in MLN (Figure 5H), the numbers of T-bet^+^IFN-γ^+^ CD4^+^ and CD8^+^ T cells reduced in the colonic epithelial tissue of IEC-*Ceacam1^–/–^* chimeras than in WT chimeras (Supplemental Figure 6D).

We also investigated the impact of host intestinal epithelial *Ceacam1* deficiency on infiltrating T cells in the colonic lamina propria. Unlike in the MLN and colonic intraepithelial compartment (Supplemental Figure 4 and Figure 7A), the percentages of IL-22^–^FoxP3^+^ Tregs in IEC-*Ceacam1^–/–^* chimeras increased compared with WT chimeras; in contrast, the percentages of IL-22^+^Fox3^–^ conventional IL-22^+^CD4^+^ T (Tcon) cells decreased (Figure 7D). In addition, very few Th/Tc1 cells were identified in IEC-*Ceacam1^–/–^* chimeras, while about 50% of the CD4^+^ and CD8^+^ T cells infiltrating the colonic lamina propria in WT chimeras had the respective Th/Tc1 phenotype (Figure 7, E and F). These results indicate that amelioration of SR-Gut-aGVHD in recipients with intestinal epithelial *Ceacam1* deficiency is associated with enrichment of IL-22^+^ pTregs in the epithelial layer and IL-22^–^ pTregs in the lamina propria of colon tissues.

### Amelioration of SR-Gut-aGVHD by Ceacam1 deficiency in IECs is associated with favorable changes in microbiota frequencies.

**Figure 8:**
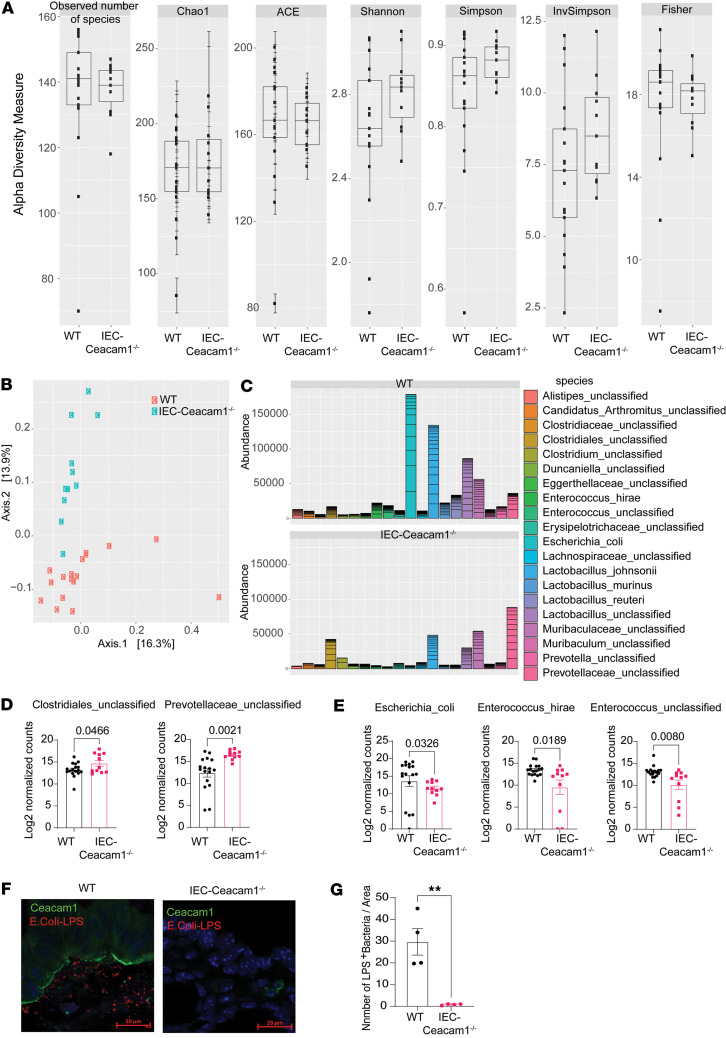
Amelioration of SR-Gut-aGVHD by *Ceacam1* deficiency in IECs is associated with favorable changes in microbiota frequencies. WT and IEC-*Ceacam1^–/–^* chimeras were engrafted with splenocytes and TCD-BM from WT C57BL/6 donors as described for Figure 2. (**A**) Mean ± SEM percentages of the diversity of ileal flora. (**B**) PCoA of the ileal flora. (**C**) The top 20 bacterial compositions at the species level. (**D** and **E**) Mean ± SEM percentages of log_2_-transformed normalized counts of Clostridiales_unclassified and Prevotellaceae_unclassified (**D**) and Escherichia_coli, Enterococcus_hirae, and Enterococcus_unclassified (**E**). (**F**) IF staining for CEACAM1 (green) and *E*. *coli*–LPS (red) in the colon. One representative photomicrograph (scale bars: 20 μm) is shown of 4 mice for each group. (**G**) Counting of LPS^+^ bacteria was performed manually using Fiji (ImageJ). LPS^+^ bacteria in 3 areas/mouse were evaluated. Statistical comparison of 4 mice/group. *n* = 11–17 from 3 experiments (**A**–**E**). *P* values are shown in **D**, **E** and **G**. **D** and **G**: *P* values were calculated by unpaired 2-tailed Student *t* tests; *P < 0.05, **P < 0.01, ***P < 0.001; **E**: Mann-Whitney *U* test.

CEACAM1^+^ IEC promotes pathogen invasion (18), notably facilitating *E*. *coli* colonization via epithelial CEACAM1 binding (31, 63). Multiple studies establish that Clostridiales, Bacteroidetes species, and *Clostridium* clusters IV/XIVa confer protection against GVHD (14, 64, 65), with Clostridiales and Prevotellaceae specifically enhancing colonic peripherally induced pTreg differentiation through TGF-β and microbial metabolites (39, 66, 67). Conversely, *Enterococcus* species and *Clostridioides difficile* correlate with aggravated GVHD pathogenesis (68–70). We thus hypothesized that IEC-specific Ceacam1 deficiency would prevent dysbiosis in SR-Gut-aGVHD. To test this, we performed ileal fecal 16S rRNA sequencing on day 25 after HCT in IEC-*Ceacam1^–/–^* and WT chimeras. The *Cecam1^–/–^* and control BALB/c breeding colonies were maintained in City of Hope Animal Resources Center. IEC-*Ceacam1^–/–^* and WT chimeras were housed separately in different cages. While α-diversity indices showed no intergroup differences (Figure 8A), principal coordinate analysis (PCoA) revealed distinct microbial clustering between IEC-*Ceacam1^–/–^* and WT chimeras (Figure 8B). In IEC-*Ceacam1*–deficient chimeras versus WT controls, we observed significantly elevated log2-normalized counts of Clostridiales and Prevotellaceae among the top 20 bacterial species, concomitant with reduced colonization by *E*. *coli*, Enterococcus hirae, and unclassified Enterococcus (Figure 8, C–E). Validating the role of epithelial CEACAM1 in facilitating *E*. *coli* invasion (63, 71), immunofluorescence (IF) analysis demonstrated prominent luminal *E*. *coli* LPS retention in WT chimeras but near-complete absence in IEC-*Ceacam1^–/–^* counterparts (Figure 8, F and G). These data collectively indicate that intestinal epithelial Ceacam1 deficiency sustained a protective microbiota profile capable of amplifying pTreg generation while concurrently abrogating enteropathogen tissue infiltration.

### Amelioration of SR-Gut-aGVHD by Ceacam1 deficiency in IECs is associated with higher numbers of antiinflammatory CD103^–^CX3CR1^hi^ MNPs and lower numbers of proinflammatory CD103^+^CX3CR1^lo^ MNPs in the colon.

**Figure 9:**
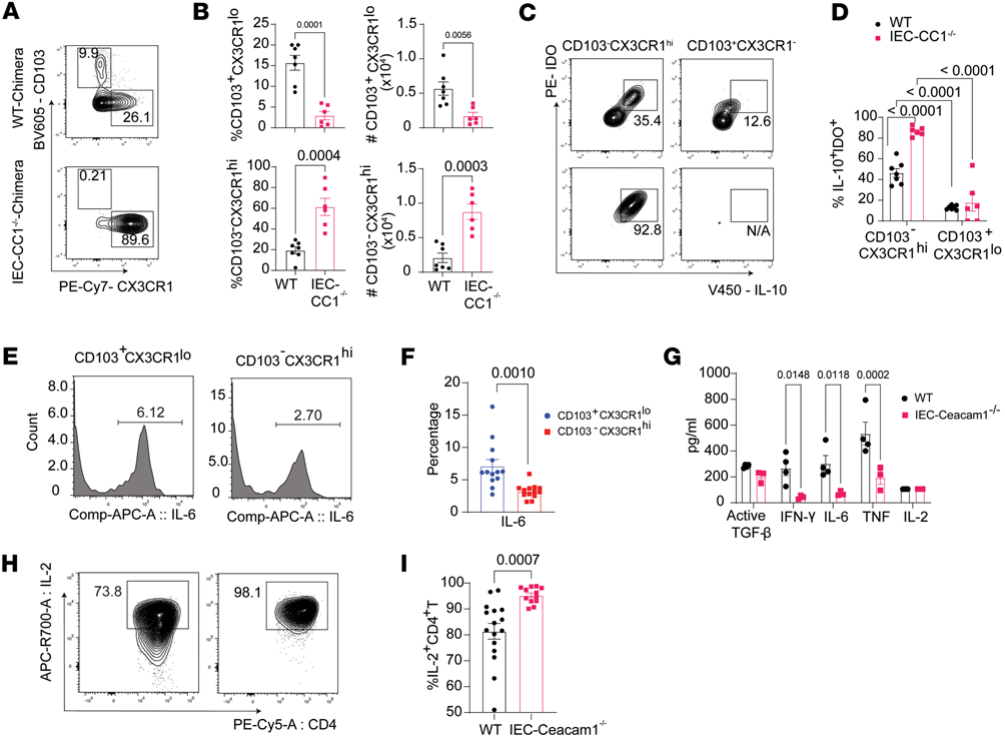
Amelioration of SR-Gut-aGVHD by *Ceacam1* deficiency in IECs is associated with higher numbers of antiinflammatory CD103^–^CX3CR1^hi^ MNPs and lower numbers of proinflammatory CD103^+^CX3CR1^lo^ MNPs in the colon. Lethally irradiated WT and IEC-*Ceacam1^–/–^* chimeric recipients were engrafted with splenocytes and TCD-BM from WT C57BL/6 donors as described for Figure 2. On day 25, CD103^+^CX3CR1^lo^ and CD103^–^CX3CR1^hi^ MNPs in the colons were analyzed, and cytokines were measured in homogenized colon tissue. (**A**) Representative flow cytometry pattern and gating strategy for CD103^+^CX3CR1^lo^ and CD103^–^CX3CR1^hi^ MNP. (**B**) Plots of mean ± SEM of %CD103^+^CX3CR1^lo^ and CD103^–^CX3CR1^hi^ MNPs among MNCs and their respective yields. *n* = 6–7. (**C**) Representative flow cytometry pattern and gating strategy showing IDO^+^IL-10^+^ population in CD103^+^CX3CR1^lo^ and CD103^–^CX3CR1^hi^ MNP. (**D**) Percentages of CD103^+^CX3CR1^lo^ and CD103^–^CX3CR1^hi^ MNPs expressing both IL-10 and IDO. Data are presented as mean ± SEM; *n* = 6–7. (**E** and **F**) Representative flow cytometry patterns and percentages of IL-6 in CD103^+^CX3CR1^lo^ and CD103^–^CX3CR1^hi^ MNPs. Data are presented as mean ± SEM; *n* = 13. Comp, compensation. (**G**) Concentration of active TGF-β, IFN-γ, IL-6, TNF, and IL-2 in colon tissue homogenate. *n* = 3–4. (**H** and **I**) Representative flow cytometry patterns and percentages of IL-2^+^CD4^+^ T cells. *n* = 12–16. Combined from 2 experiments. *P* values are shown in **B**, **D**, **F**, **G**, and **I**. Unpaired 2-tailed Student’s *t* test was used to compare 2 means in **B**, **F**, and **I**. Two-way ANOVA was used to compare means in **D** and **G**.

Donor CD103^–^CX3CR1^hi^ MNPs play a protective role in the setting of SR-Gut-aGVHD (17). CX3CR1^hi^ MNPs keep close contact with IECs to mediate clearance of enteropathogens and commensal bacteria that invade epithelial barriers (72). In the current study, we found that numbers of CD103^–^CX3CR1^hi^ MNPs were higher in IEC-*Ceacam1^–/–^* than in WT chimeras, while numbers of CD103^+^CX3CR1^lo^ MNPs were lower in IEC-*Ceacam1^–/–^* than in WT chimeras (Figure 9, A and B). Moreover, CD103^–^CX3CR1^hi^ MNPs produced more IDO and IL-10 than CD103^+^CX3CR1^lo^ mononuclear cells (MNCs) (Figure 9, C and D). In contrast, CD103^+^CX3CR1^lo^ DCs produced significantly higher levels of IL-6 than did CD103^–^CX3CR1^hi^ MNPs (Figure 9, E and F).

In addition to the reduction of IL-6, IL-2 is an important cytokine for promoting pTreg development in the presence of TGF-β (73–76). Therefore, we hypothesized that the expansion of pTregs in IEC-*Ceacam1^–/–^* recipients was also associated with enhanced IL-2 production. IL-2 is primarily produced by CD4^+^ T cells, while TGF-β is mainly produced by fibroblasts and epithelial cells in the colon (77–79). To investigate the cytokines in the intestinal environment, we measured active TGF-β, IFN-γ, IL-6, TNF-α, and IL-2 in homogenized colon tissue of WT and IEC-*Ceacam1^–/–^* chimeras. IFN-γ, IL-6, and TNF-α concentrations were lower in IEC-*Ceacam1^–/–^* than in WT chimeras. We found no difference in active TGF-β or IL-2 concentrations between the IEC-*Ceacam1^–/–^* and WT chimeras (Figure 9G), but the percentages of IL-2^+^CD4^+^ T cells in the intraepithelial colon epithelium were higher in IEC-*Ceacam1^–/–^* chimeras than in WT chimeras (Figure 9, H and I). Collectively, the results indicate that IEC Ceacam1 deficiency promoted expansion of IL-10/IDO–producing CD103^–^CX3CR1^hi^ regulatory MNPs that drive pTreg generation, while attenuating proinflammatory CD103^+^CX3CR1^lo^ MNPs secreting IL-6/TNF-α that potentiate pathogenic Th1/Th17 differentiation.

## Discussion

Patients with SR-Gut-aGVHD have poor prognosis and 75%–90% mortality due to poor understanding of its pathogenesis (4, 80). A previously reported murine model of SR-GVHD indicated that donor T cell–independent mechanisms might play more prominent roles in the pathogenesis of SR-GVHD than was considered previously, because there was no difference in the percentage of Th1 or Th17 cells in SR- and non-SR-GVHD recipients; however, Th/Tc22 cells in the gut tissues were not evaluated (81). With another murine model, we recently reported that SR-Gut-aGVHD attributed to dysbiosis resulted from expansion of IL-22–producing CD4^+^ and CD8^+^ T cells and loss of protective CX3CR1^hi^ MNPs, but the pathogenesis process remains unclear. In the current studies, with IMC analysis of colon biopsy samples from 3 SR-Gut-aGVHD and 3 non-SR-Gut-aGVHD patients, we observed that, consistent with results in murine models, higher numbers of IL-22–producing CD4^+^ and CD8^+^ T cells were present in the SR-Gut-aGVHD tissue sections. Colonic epithelial cells of both human and murine SR-Gut-aGVHD recipients upregulated expression of CEACAM1, a receptor that can mediate bacterial translocation from the lumen to the tissue (31). In murine models, combined scRNA-Seq and ATAC-Seq analysis and high-dimensional flow cytometry showed that *Ceacam1* deficiency in IECs effectively prevented SR-Gut-aGVHD pathogenesis in association with a reduction of bacterial translocation and reversal of dysbiosis; as well as with reduced terminal differentiation and survival of Th1, Th2, and Th17 in the MLNs, lower frequencies of IL-22^+^RORγt^+^ Th/Tc17 or IL-22^+^AHR^+^ Th/Tc22 cells in the MLN and colon tissues, but higher frequencies of IL-22^+^FoxP3^+^RORγt^–^AHR^–^CD4^+^ Tregs. These observations provide insights into SR-Gut-aGVHD pathogenesis.

First, intestinal epithelial *Ceacam1* deficiency prevents dysbiosis in steroid-treated recipients with GVHD. Bacterial binding to intestinal epithelial CEACAM1 facilitates bacterial adhesion and invasion into gut tissue (18). Consistently with this finding, we observed high expression of CEACAM1 by colonic epithelial cells in mice and humans with SR-Gut-aGVHD. *Ceacam1* deficiency in the colonic epithelial cell prevented *E*. *coli* adhesion to the epithelium and invasion into tissues. In addition, some of the IL-22^+^CD4^+^ T cells became IL-22^+^FoxP3^+^ Tregs that were enriched in the intraepithelial compartment. These cells may augment IEC regeneration (82) and help maintain cellular integrity that reduces bacterial invasion. Lower production of IL-22 by reduction of pathogenic Th17 and Th22 cells associated with IEC-*Ceacam1* deficiency may also help to prevent dysbiosis (17).

Intestinal epithelial *Ceacam1* deficiency alters the balance between antiinflammatory CD103^–^CX3CR1^hi^ MNPs and proinflammatory CD103^+^CX3CR1^lo^ MNPs in steroid-treated recipients with GVHD. Pathogen invasion and bacterial translocation can turn antiinflammatory CX3CR1^hi^ MNPs into proinflammatory CX3CR1^lo^ MNPs (83, 84). We observed that, compared with WT controls, the colon of steroid-treated IEC-*Ceacam1*–deficient recipients with GVHD had higher numbers of CX3CR1^hi^ MNPs that produced IL-10 and IDO but lower numbers of CX3CR1^lo^ MNPs that produced IL-6 and TNF-α. Activated donor-type colonic CD103^+^CD11c^+^ DCs played an important role in regulating the severity of aGVHD (85). Antiinflammatory CX3CR1^hi^ MNPs that produce IL-10 and IDO may inhibit colonic DC activation and augment pTreg expansion and function in the tissues. IL-10 acts on Tregs to maintain expression of Foxp3 and their suppressive function (86), and IDO promotes pTreg differentiation and blocks their conversion into Th17-like T cells (87). Antiinflammatory effects of GCs were reported to be mediated through IL-10–dependent Treg activity (88).

Intestinal epithelial *Ceacam1* deficiency alters the balance between Th effectors and Tregs in steroid-treated recipients with GVHD. The lower numbers of terminally differentiated Th subsets and the expansion of intermediate CD4^+^ T cells in the MLN of steroid-treated IEC-*Ceacam1*–deficient recipients with GVHD were associated with lower TCR, IL-2/Stat5, IL-6/Stat3, and TLR signaling and higher activity in the glycine-serine/threonine and valine-leucine/isoleucine pathways. These differences may have resulted from changes in microbiome profiles and their metabolites and changes in the activation status of CX3CR1^hi^ MNPs and CD103^+^ DCs. LPS from pathogens can augment TLR signaling in DCs and T cells, as well as augmenting DC maturation and T cell activation and differentiation (42, 43). Metabolites from serine and leucine augment effector T cell proliferation (44) and sustain mTORC1 activation and functional programming of pTregs (45). Consistently with these findings, we observed that a portion of IL-22^+^CD4^+^ T cells were transdifferentiated into IL-22^+^FoxP3^+^CD4^+^ T cells. The decreased pathogen invasion and augmentation of beneficial commensal bacterial in intestinal epithelial *Ceacam1*–deficient recipients treated with steroids could account for the lower production of pathogenic Th subsets and higher production of pTregs as compared with WT controls, consistent with findings reported by others (67, 89). Therefore, in SR-Gut-aGVHD, the reduction in terminal differentiation and survival of proinflammatory Th1, Th2, and Th22 cells contributed to prevention of the disease, while a small percentage of IL-22^+^ pTregs may also have played an important role.

Intestinal epithelial *Ceacam1* deficiency enriches IL-22^+^FoxP3^+^ Tregs in the intestine epithelial compartment without causing dysbiosis in steroid-treated recipients with GVHD. Under inflammatory conditions, IL-22 augments pathogen colonization and dysbiosis in gut tissues (17), and overproduction of IL-22 leads to dysbiosis and SR-Gut-aGVHD in aGVHD recipients (17). It was also recently reported that prolonged administration of steroid directly augmented immune-mediated damage of intestinal epithelial regeneration and contributed to SR-Gut-aGVHD pathogenesis (36). *Ceacam1* deficiency in IECs effectively prevented SR-Gut-aGVHD but did not affect acute gut GVHD in the absence of steroid treatment. Acute gut GVHD is mediated by alloreactive lymphocytes, while SR-Gut-aGVHD is mediated mainly by dysbiosis with invasion of pathogens and loss of antiinflammatory CX3CR1^hi^ MNPs (17). In the absence of pathogen invitation and bacterial translocation by CEACAM1 on IECs, enrichment of IL-22^+^CD4^+^ Tregs in the intraepithelial compartment did not cause dysbiosis and instead may have augmented IEC regeneration and integrity, thereby inhibiting dysbiosis. Thus, targeting CEACAM1 on IECs during steroid treatment may offer a potential approach toward augmenting intestinal epithelial regeneration by increasing the numbers and function of IL-22–producing Tregs.

Ceacam1 interacts with Ceacam1 in a homophilic fashion (18), as well as interacting with Tim3 and Glectin 9 on immunocytes to deliver inhibitory signaling to the immunocytes (90). Ceacam1 on IECs mediate bacterial translocation (18). We observed that, consistent with a previous report (28), in the absence of steroid treatment, Ceacam1 deficiency on donor cells or recipient cells appeared to augment acute gut GVHD. However, in the presence of steroid resistance and dysbiosis, Ceacam1 deficiency on IECs resulted in prevention of acute SR-Gut-GVHD, with reduced bacterial translocation and an increase in regulatory CX3CR1^hi^ MNPs, as well as with reduced infiltration of proinflammatory Th1 and Th17 cells, but an increase of IL-22^+^ pTregs, although the mechanisms remain unclear. Therefore, due to the pleiotropic effects of CEACAM1, targeting Ceacam1 systemically may result in augmentation of inflammation; however, in the inflammatory intestinal diseases with dysbiosis (e.g., SR-Gut-GVHD), local blockade of Ceacam1 may prevent bacterial translocation and ameliorate the disease. In our preliminary studies, we observed that rectal delivery of Ceacam1 peptides but not anti-Ceacam1 prevented SR-Gut-GVHD in murine models.

In addition, with IMC analysis of patient colon biopsy tissue sections, we observed that all IL-22^+^CD8^+^ T cells expressed Ahr, and some IL-22^+^CD8^+^ T cells also expressed T-bet, although this varied from patient to patient. We also observed that there were variable percentages of IL-22^+^IFN-γ^+^IL-17A^+^Ahr^+^T-bet^+^Rorc^+^ T cells in patients with SR-Gut-GVHD but not control patients.

It was previously reported that induction of Tc22 cells in tumor tissue required expression of Ahr and repression of Tbx21 (91). It was also reported that ornithine decarboxylase (ODC) deficiency in donor T cells can cause T cell lineage infidelity, in which ODC*^–/–^* T cells could become Th1, Th2, Th17, or Th22 under a Th1 culture condition or environment (92); and steroid treatment downregulated expression of ODC (93). Our data suggest the possibility of T cell lineage infidelity in SR-GVHD tissues. Future studies are needed to address this issue.

In summary, we demonstrated that *Ceacam1* deficiency in IECs effectively prevented SR-Gut-aGVHD. Based on the current observations, we propose that the key initial effect of *Ceacam1* deficiency in IECs is to block pathogen invasion and bacterial tissue translocation while preventing dysbiosis. In WT recipients, *E*. *coli* pathogen invasion and commensal bacterial tissue translocation made antiinflammatory CX3CR1^hi^ MNPs become proinflammatory CD103^+^CX3CR1^lo^ MNPs that activated proinflammatory tissue DCs. These migrated into the MLN to activate and mediate donor T cell activation and differentiation into pathogenic Th1, Th17, and Th22 cells; in turn, those pathogenic Th cells infiltrated gut tissues and attracted infiltration of other leukocytes, leading to full-blown SR-Gut-aGVHD. In contrast, *Ceacam1* deficiency in IECs prevented *E*. *coli* pathogen invasion and reduced bacterial tissue translocation, as well as increasing beneficial commensal bacteria, resulting in maintenance of antiinflammatory CX3CR1^hi^ MNPs. This not only reduced activation of tissue DCs and reduced activation and terminal differentiation of pathogenic Th1, Th2, Th17, and Th22 cells, but also augmented differentiation and transdifferentiation to generate peripheral FoxP3^+^CD4^+^ pTregs. In the intestine, CX3CR1^hi^ MNPs and beneficial commensal bacterial further augmented expansion of pTregs, and the pTregs not only effectively prevented local inflammation but also may have augmented IEC regeneration, leading to effective prevention of SR-Gut-aGVHD. Therefore, targeting CEACAM1 expressed by IECs may represent a promising approach to prevent SR-Gut-aGVHD.

## Methods

Further information is presented in Supplemental Methods.

### Sex as a biological variable.

Analyses included both male and female patients and animals. Sex was not explicitly considered as a variable in analyses.

### Mice.

WT BALB/c (H-2^d^) and C57BL/6 (H-2^b^) mice were purchased from the National Cancer Institute (NCI). CD45.1 BALB/c mice and *Villin^Cre^* mice were purchased from the The Jackson Laboratory. *Ceacam1^fl/fl^* breeders were provided by Sonia M. Najjar (Ohio University). All mice were maintained in a pathogen-free room in City of Hope Animal Research Center.

### Methods.

Induction and assessment of SR-Gut-GVHD (17), measurement of cytokines in tissue homogenization (94), histopathology, histoimmunofluorescence staining, and IMC (17, 34, 95, 96), as well as scRNA-Seq combined with ATAC-Seq using the 10x Genomics chromium platform (17, 97), microbiome analysis using StrainID 16s kit with PacBio long reads (17), and statistical analysis, are described in previous publications (95, 96, 98) and/or Supplemental Methods. Additional animal model details are provided in Supplemental Methods.

### Antibodies.

mAbs specific for CD45.2 (clone 104), CD8 (53-6.7), IL-10 (JES5-16E3), IL-17A (TC11-18H10), IL-2 (JES6-5H4), CEACAM-1 (CC1), and IL-6 (MP5-20F3) were purchased from BD Biosciences. mAbs specific for PD-1 (J43), FOXP3 (FJK-16s), AHR (4MEJJ), RORγt (AFKJS-9), and CD103 (2E7) were purchased from Invitrogen. mAbs specific for CD4 (RM4-5), H-2Kb (AF6-88.5), CD45.1 (A20), neuropilin-1 (3E12), TCR-β (H57-597), Helios (22F6), IFN-γ (XMG1.2), T-bet (4B10), TNF-α (MP6-XT22), IL-22 (Poly5164), IL-10 (JES5-16E3), CCR6 (29-2L17), CX3CR1 (SA011F11), and CD11c (N418) were purchased from BioLegend along with the hybridomas for anti–PD-1 conjugated with Super Bright 780, anti–PD-1 conjugated with IL-10, and anti-CCR6 conjugated with Dazzle 594. Flow cytometry analyses were performed with Cytek Aurora, and the resulting data were analyzed with FlowJo software V10 (Tree Star).

### Statistics.

Student’s unpaired *t* test was used to compare 2 groups when data were normally distributed. Mann-Whitney *U* test was used to compare 2 groups when data were not normally distributed. For comparing more than 2 groups, Kruskal-Wallis test with Dunn’s multiple-comparison test was used was used to compare 2 groups when data were not normally distributed. One-way ANOVA with Tukey’s multiple-comparison test, with the Greenhouse-Geisser correction or Student’s *t* test corrected for multiple comparisons using the Holm-Šídák method, were used to compare means in paired samples. Ordinary 1-way ANOVA with Tukey’s correction for multiple comparisons or 2-way ANOVA with Tukey’s or Šídák’s correction for multiple comparisons was used to compare means in unpaired samples. Log-rank test was used for survival comparisons. Nonlinear regression (curve fit) was used for body weight change and diarrhea development comparisons. All statistical analyses were performed using GraphPad Prism 8. *P* values less than 0.05 were considered statistically significant.

### Study approval.

All patient samples were collected after approval of the retrospective tissue study by the City of Hope Institutional Review Board (IRB20509). All animal experiments were approved by the City of Hope IACUC (IACU03008).

### Data availability.

16S PacBio SMRT Sequencing data have been deposited in the NCBI’s Gene Expression Omnibus database (GEO GSE159031). 16S MiSeq data have been deposited in the GEO database (GSE159418). The 2 datasets were combined in a superseries (GEO GSE159419). All other data supporting the findings of this study are available within the article and its supplemental files or from the corresponding author upon reasonable request. Values for all data points in graphs are reported in the Supporting Data Values file.

## Author contributions

QS designed and performed experiments and wrote the manuscript. MZ performed IMC, IF staining, and data analysis. QL and BL performed experiments. XW, HQ, and THK performed PacBio 16S RNA-Seq, scRNA plus ATAC-Seq, and data analysis. MK and JS provided *Ceacam1^–/–^* BALB/c mice and anti-CEACAM1 mAb, and JS also provided critical review of the manuscript. RKP, JLL, and RN provided human colon biopsy samples. PJM provided advice on experimental design and critically reviewed and edited the manuscript. DZ supervised the research and wrote the manuscript.

## Supplementary Material

Supplemental data

Supporting data values

## Version 1 09/09/2025

Electronic publication
