# Supplementary material for: Intestinal epithelial Ceacam1 deficiency prevents steroid-refractory acute gut graft-versus-host disease
Source: JCI Insight. 2025 Sep 9;10(17):e186984. doi: 10.1172/jci.insight.186984 (PMC12487693; doi:10.1172/jci.insight.186984)
Supplement: Supplemental data [file jciinsight-10-186984-s289.pdf]

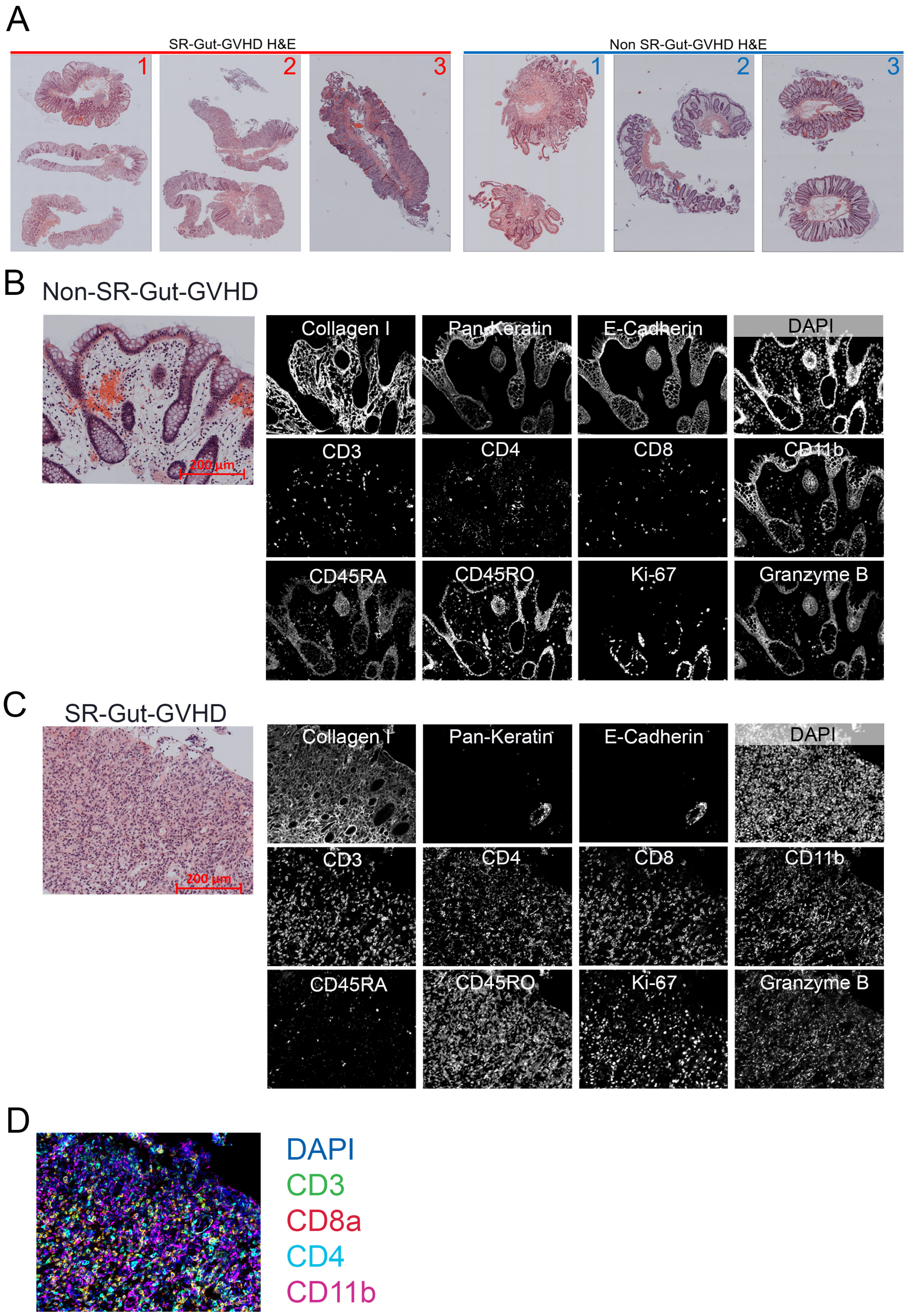
A
SR-Gut-GVHD H&E
Non SR-Gut-GVHD H&E
1
2
3
1
2
3
B
Non-SR-Gut-GVHD
200 µm
Collagen I
Pan-Keratin
E-Cadherin
DAPI
CD3
CD4
CD8
CD11b
CD45RA
CD45RO
Ki-67
Granzyme B
C
SR-Gut-GVHD
200 µm
Collagen I
Pan-Keratin
E-Cadherin
DAPI
CD3
CD4
CD8
CD11b
CD45RA
CD45RO
Ki-67
Granzyme B
D
DAPI
CD3
CD8a
CD4
CD11b

Fig. S1: IMC analysis of immune cell infiltration in colon biopsy tissue of SR-GVHD and Non-SR-GVHD patients. (A) Representative H&E staining images of colon biopsies from SR-Gut-GVHD and Non-SR-GVHD patients. **(B)** Representative H&E staining image and IMC patterns in colon biopsy from 1 of 3 patients with Non-SR-Gut-GVHD, showing expression of the indicated stromal markers, immune markers, Ki-67, Granzyme B and DNA by the cells. **(C)** Representative colon image mass cytometry patterns from 1 of 3 patients with SR-GVHD, showing expression of the indicated stromal markers, immune markers, Ki-67, Granzyme B and DNA by the cells. Scale bar (200μm). **(D)** Representative mass cytometry merged image of DAPI (blue), CD3 (green), CD8a (red), CD4 (cyan), and CD11b (magenta).

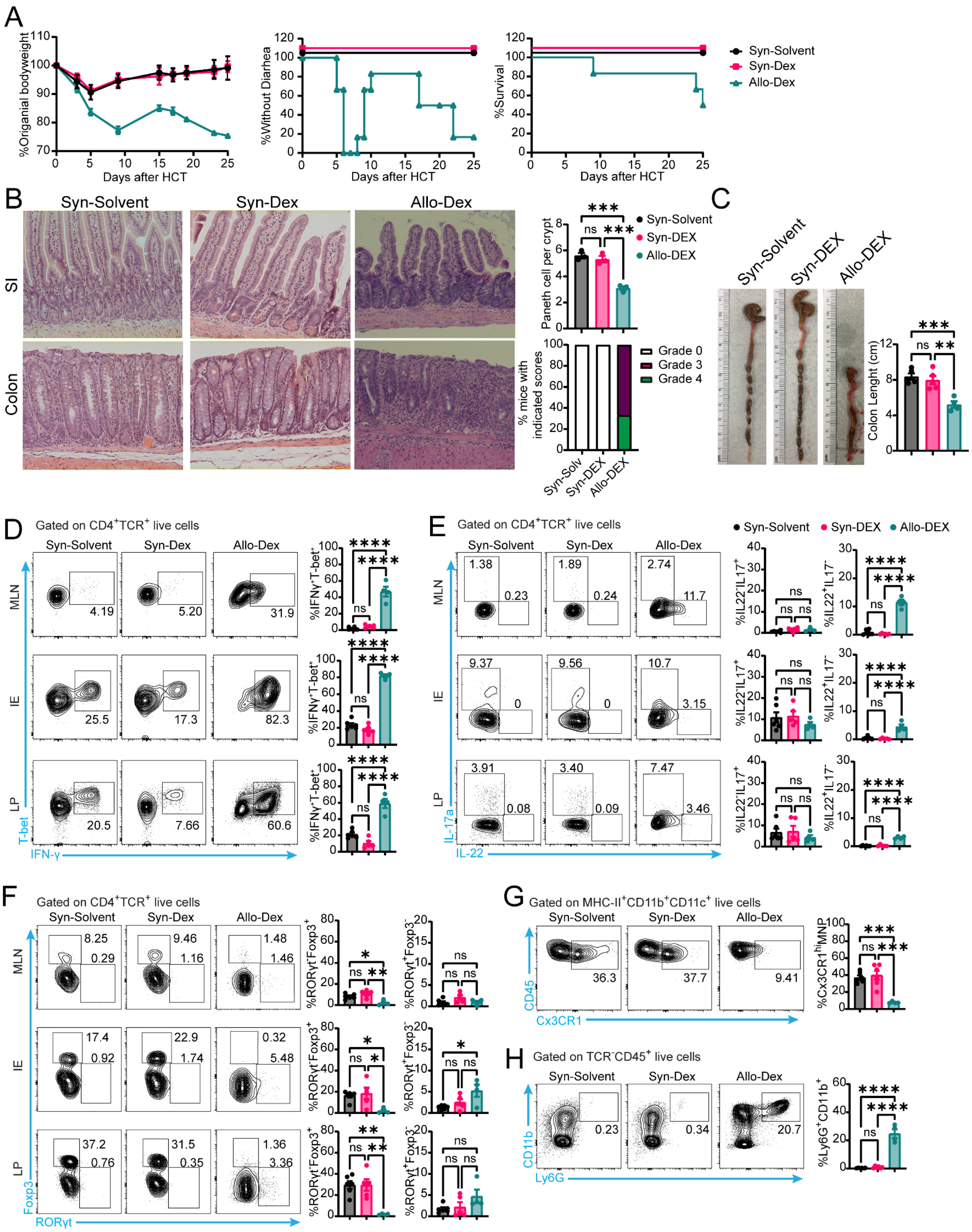

Fig. S2. Syngeneic transplant with prolonged DEX exposure does not induce gut lesions. Lethally irradiated WT BALB/c recipients were given T cell depleted bone marrow cells (TCD-BM, 2.5x10^6) with or without splenocytes (2.5x10^6) from BALB/c or C57BL/6 donors. Recipients were

given four total injections of dexamethasone (4-DEX) or PBS (No-DEX) on days 3, 10, 15 and 20 after HCT. Data was collected on Day 25 after HCT. **(A)** Plots of %Original bodyweight, %mice without diarrhea, and %Survival is shown. n= 6 from two replicate experiments. **(B)** Histopathology of small intestine and colon was evaluated. Representative micrographic photos of small intestine and colon (original magnification, x200) are shown, along with means ± SEM of the numbers of Paneth cell per crypt in the small intestine and % mice with indicated histopathological scores in the colon. n=3 per group. **(C)** colon lengths are shown. n= 5 from two replicate experiments. **[D-F]** Representative flow cytometry patterns and means ± SEM of **(D)** % IFN-γ^{+}T-bet^{+}, **(E)** % IL-22^{-}IL-17^{+} and % IL-22^{+}IL-17^{-}, **(F)** % RORγt^{-}Foxp3^{+} and % RORγt^{+}Foxp3^{-}, among CD4 subsets from MLN, IEL, and LPL of the colon. **[G-H]** Representative flow cytometry patterns and means ± SEM of **(G)**% CX3CR1^{hi} MNP, **(H)** % Ly6G^{+}CD11b^{+} in colon LPL. n = 4-6, two replicates. Each dot represents one mouse. *P* value was determined by unpaired two-tailed Student' t test. NS, $P \geqslant 0.05$; *$P < 0.05$.

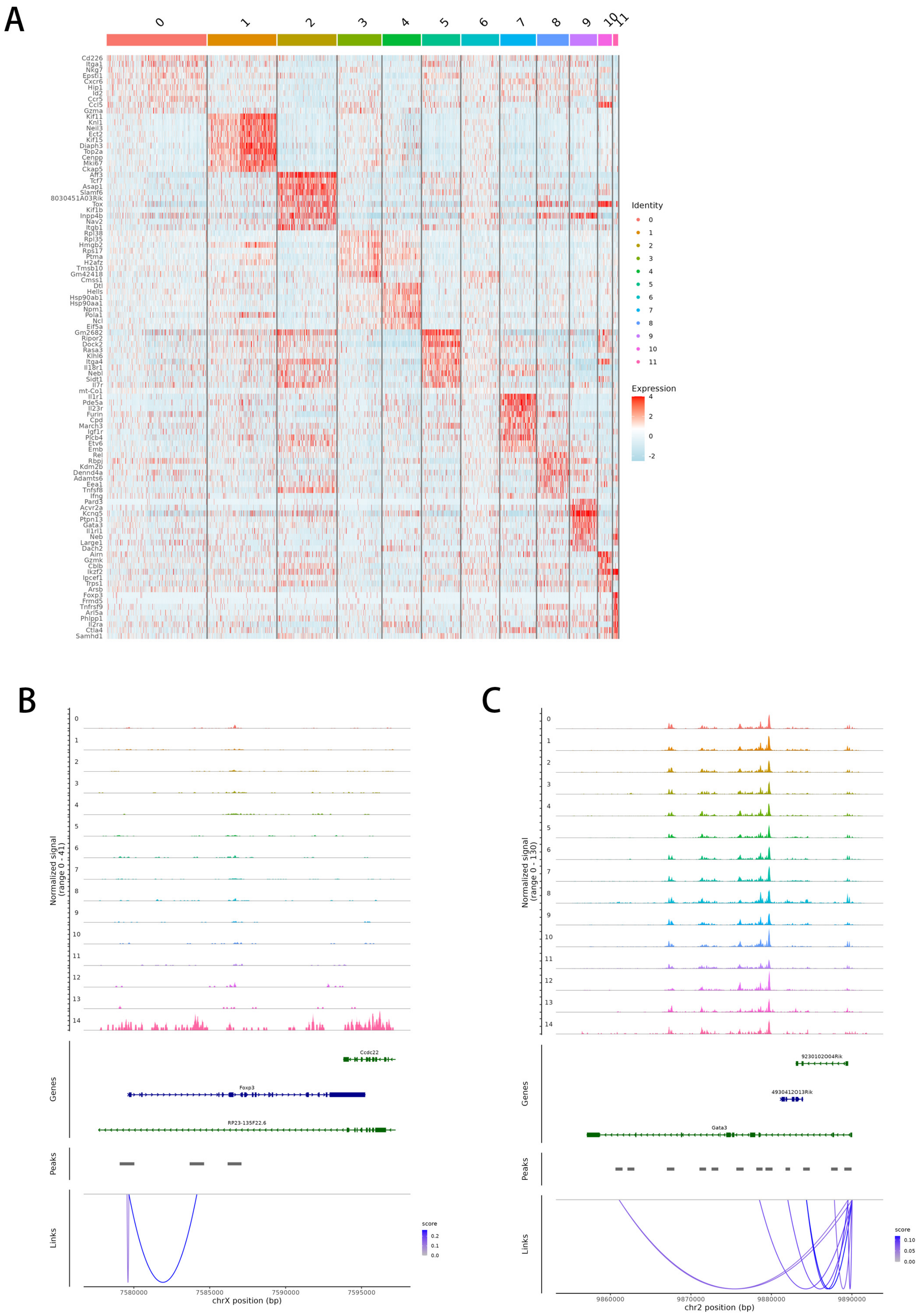
A
B
C
Identity
Expression
Normalized signal (range 0 - 41)
Normalized signal (range 0 - 130)
Genes
Peaks
Links
score
Ccdc22
Foxp3
RP23-135F22.6
9230102O04Rik
4930412O13Rik
Gata3
chrX position (bp)
chr2 position (bp)

Fig. S3: scRNA+ATAC Seq reveals distinct subpopulations and transcriptional signatures of $CD4^+$ T cells in MLN of IEL-$CC1^{-/-}$ chimeras and WT chimeras. **(A)** Heatmap showing differentially expressed coding genes for the 15 clusters in Figure 4A. **(B)** Chromatin accessibility for Foxp3. **(C)** Chromatin accessibility for Gata3.

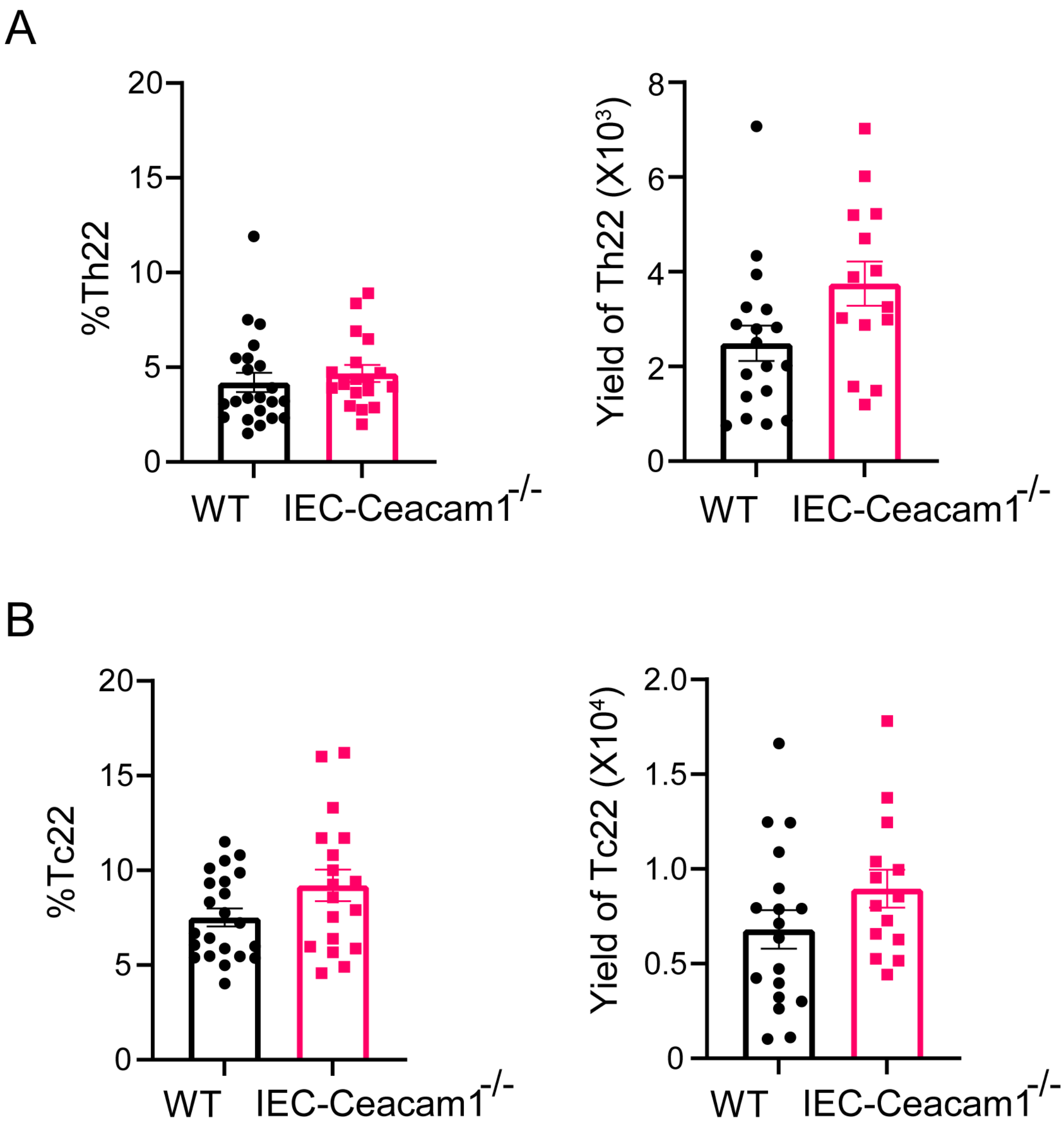

Fig. S4: *Ceacam1* deficiency on host intestinal epithelia cells did not affect the numbers of Th/Tc22 cells in the mesenteric lymph node (MLN). WT chimeras and IEC-*Ceacam1*$^{-/-}$ chimeras were engrafted with splenocytes and TCD-BM from WT C57BL/6 donors and treated with 4-DEX as described in Fig.2. **(A-B)** Means ± SEM of % among $CD4^+$ cells and yields of IL-22^+IL17A$^-$$CD4^+$ (Th22) (A) and IL-22^+IL17A$^-$$CD8^+$ T (Tc22) (B) cells in MLN. n=22 (%WT), 18 (%IEC-*Ceacam1*$^{-/-}$), 18 (Yield of WT), 14 (Yield of IEC-*Ceacam1*$^{-/-}$). Combined from three replicate experiments. Unpaired two-tailed Student's t-test was used to compare means.

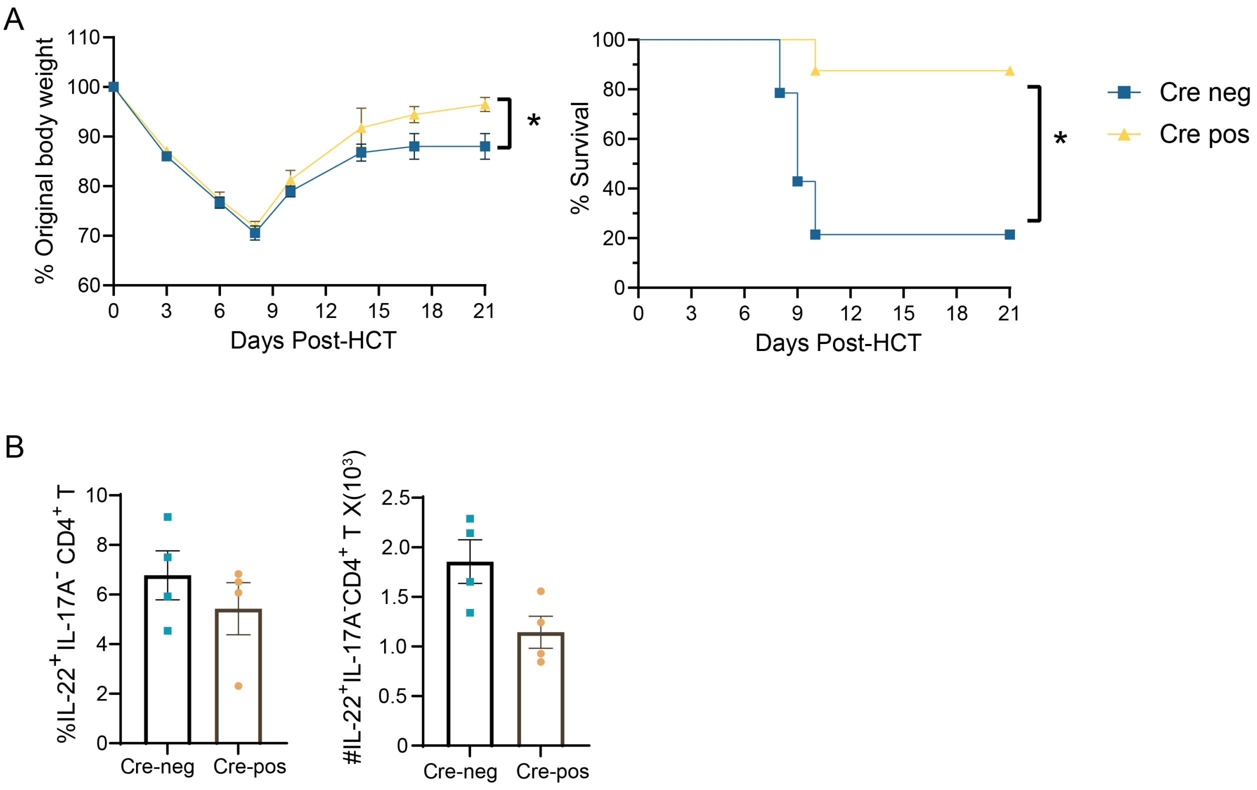

Fig. S5: *Ceacam1* deficiency in host intestinal epithelial cells does not affect the numbers of IL-22$^+$IL-17A$^-$CD4$^+$T cells in MLN in the BALB/c donor to C57BL/6 recipient model. VillinCre-pos-*Ceacam1*$^{fl/fl}$ and VillinCre-neg-*Ceacam1*$^{fl/fl}$ C57BL/6 recipients were engrafted with splenocytes together with TCD-BM from CD45.1 BALB/c donors and treated with 4-DEX on days 3, 10, 15 and 20 after HCT. **(A)** Plots of %Original bodyweight and %survival of Villin-Cre *Ceacam1*$^{fl/fl}$ mice(Cre pos) and control mice (Cre neg). n=14 (Cre neg), 8 (Cre pos) from two replicate experiments. Nonlinear regression (curve fit) was used for body weight comparisons. Log-rank test was used for survival comparison. **(B)** On day 21, mesenteric lymph nodes from Cre-pos or Cre-neg recipients were collected to analyze T cell subsets. Means ± SEM of % among CD4$^+$ cells and yields of IL-22$^+$IL-17A$^-$CD4$^+$ T cells.

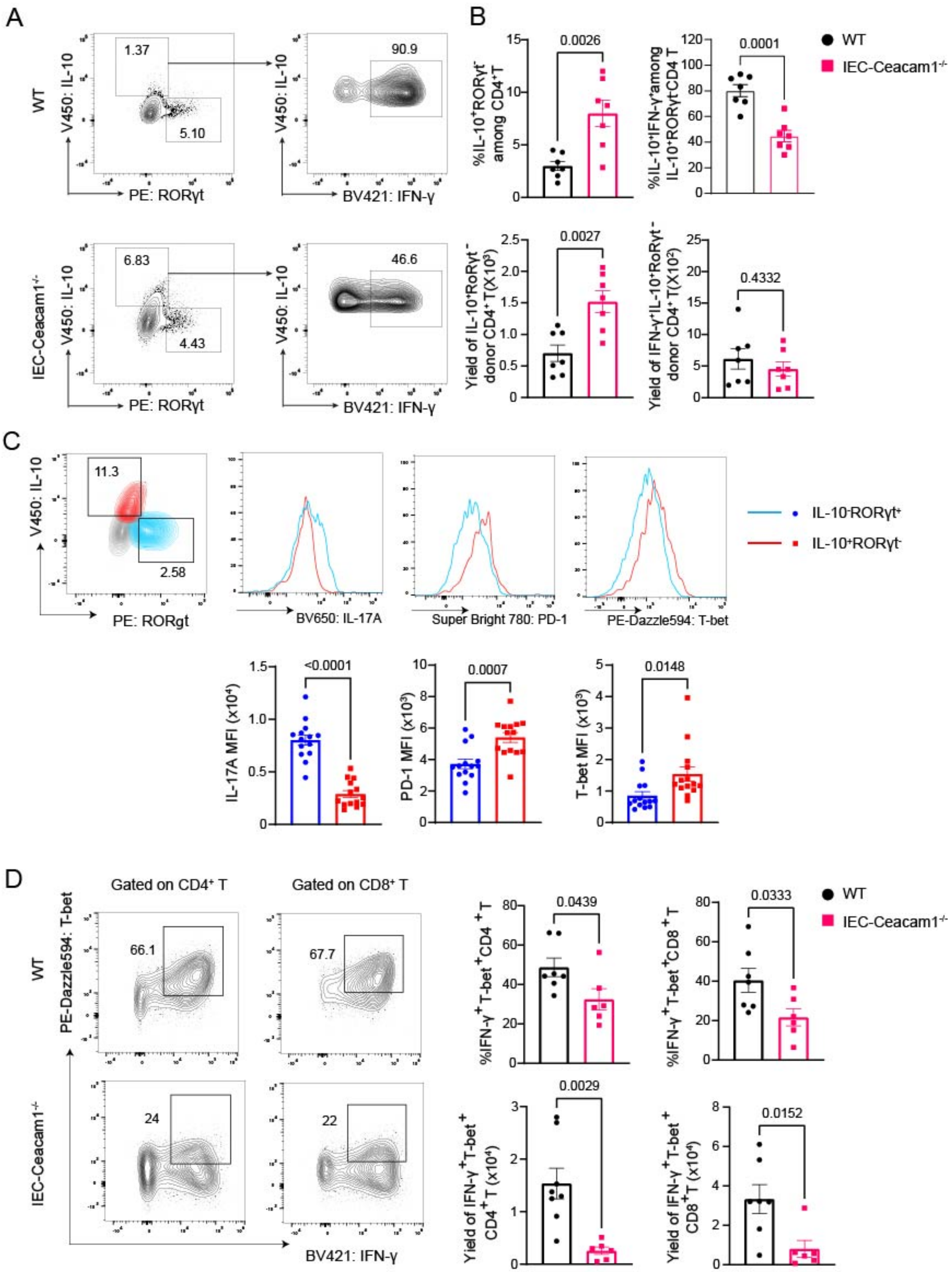
A
B
WT
IEC-Ceacam1-/-
V450: IL-10
PE: RORγt
BV421: IFN-γ
1.37
5.10
90.9
6.83
4.43
46.6
%IL-10+RORγt- among CD4+T
%IL-10+IFN-γ+among IL-10+RORγt-CD4 T
Yield of IL-10+RoRγt- donor CD4+ T(X10³)
Yield of IFN-γ+IL-10+RoRγt- donor CD4+ T(X10²)
0.0026
0.0001
0.0027
0.4332
C
PE: RORgt
11.3
2.58
BV650: IL-17A
Super Bright 780: PD-1
PE-Dazzle594: T-bet
IL-10-RORγt+
IL-10+RORγt-
IL-17A MFI (x10⁴)
PD-1 MFI (x10³)
T-bet MFI (x10³)
<0.0001
0.0007
0.0148
D
Gated on CD4+ T
Gated on CD8+ T
PE-Dazzle594: T-bet
66.1
67.7
24
22
%IFN-γ+T-bet+CD4+T
%IFN-γ+T-bet+CD8+T
Yield of IFN-γ+T-bet+ CD4+ T (x10⁴)
Yield of IFN-γ+T-bet+ CD8+T (x10⁴)
0.0439
0.0333
0.0029
0.0152

Fig. S6: Amelioration of SR-Gut-aGVHD by Ceacam1 deficiency in the host is associated with in higher numbers of pTregs but lower numbers of Th/Tc1 cells in the colonic epithelium. WT chimeras or IEC-Ceacam1-/- chimeras were engrafted with splenocytes together with TCD-BM from WT C57BL/6 donors as described in Fig.2. **(A-B)** Representative flow cytometry patterns and Means ± SEM of % and yield of RORγt$^-$IL-10+ among CD4$^+$ T cells and IFN-γ+IL-10+ among RORγt$^-$IL-10+ CD4$^+$ T cells in the colonic epithelium are shown. n=7. **(C)** Representative flow cytometry patterns and Means ± SEM of MFI of IL-17A, PD-1 and T-bet expression in the RORγt$^+$IL-10$^-$ and RORγt$^-$IL-10$^+$CD4$^+$ T cells among colon intraepithelial cells. n=14. (D) Representative flow cytometry patterns and Means ± SEM of % and yield of T-bet$^+$IFN-γ$^+$ CD4$^+$ and CD8$^+$ T cells among colon intraepithelial cells are shown. n=7. Combined from two replicate experiments. Unpaired two-tailed Student's t test was used to compare two means.

Supplemental Materials and Methods

Patient sample information

The clinical database was searched for patients who underwent hematopoietic stem cell transplantation at City of Hope and developed gut GVHD. Colon biopsy samples were classified as mild or severe GVHD based on clinical, endoscopic and pathologic criteria. Histology grading was based on increased crypt apoptosis (grade 1), apoptosis with crypt abscess (grade 2), individual crypt necrosis (grade 3) and diffuse denudation of areas of mucosa (grade 4). Samples from patients with GVHD who did not respond to steroid treatment were classified as steroid resistant GVHD (SR-GVHD). This retrospective study was approved by the COH Institutional Review Board. Since samples were anonymized based on IRB requirements, we were provided with tissue sections labeled with SR-GVHD (severe GVHD) or Non-SR-GVHD (mild GVHD) without any more details.

Induction and assessment of GVHD

In general, male mice were used at 8 to 12 weeks of age, BALB/c recipients were exposed to 850 cGy total body irradiation in a single fraction, and C57BL/6 recipients were exposed to 1300 cGy total body irradiation in a single fraction. Splenocytes and T cell depleted bone marrow cells from donors were injected via tail vein into recipients 6-8 hours after irradiation. Dexamethasone (5mg/kg) was given by i.v. injection on days 3, 10, 15 and 20 after HCT. Depletion of T cells from the bone marrow was accomplished by using biotinconjugated anti-CD4 and anti-CD8 mAbs, and streptavidin Microbeads (Miltenyi Biotec, Germany), followed by passage through an autoMACS Pro cell sorter (Miltenyi Biotec, Germany). Clinical acute gut GVHD was assessed by diarrhea, body weight and survival [1].

Establish bone marrow chimera mice

Ceacam1$^{-/-}$ BALB/c mice were exposed to 850 cGy total body irradiation (TBI) with the use of a [137Cs] source 8 hours before HCT, and then given T cell-depleted BM (TCD-BM) cells (10x 10^6) from WT BALB/c mice by tail vein injection to establish IEC-Ceacam1$^{-/-}$-Chimeras. WT BALB/c mice were exposed to 850 cGy total body irradiation (TBI) with the use of a [137Cs] source 8 hours before HCT, and then given T cell-depleted BM (TCD-BM) cells (10x 10^6) from Ceacam1$^{-/-}$ BALB/c mice by tail vein injection to establish HC-Ceacam1$^{-/-}$-Chimeras. WT BALB/c mice were exposed to 850 cGy total body irradiation (TBI) with the use of a [137Cs] source 8 hours before HCT, and then given T cell-depleted BM (TCD-BM) cells (10x 106) from WT BALB/c mice by tail vein injection to establish WT-Chimeras as control.

Isolation of cells from mesenteric lymph node and large Intestine

Lymph nodes were mashed through a 70 µm cell strainer, and mononuclear cells (MNC) were isolated from the cell suspensions with Percoll. Large intestine was cut first longitudinally and then laterally into pieces of approximately 0.5 cm length. Tissue pieces were incubated with 20 mL of pre-digestion solution (1× HBSS) containing 5 mM EDTA, 5% fetal bovine serum (FBS), 1 mM DTT) for 20 minutes at 37 °C under continuous shaking, then passed through 100 µm strainer and held for at least 10 minutes on ice, Intestine epithelial lymphoid cells in the supernatant were collected, repeat this process twice. Then tissue pieces were digested with enzyme to isolate the lamina propria cells, following the protocol of Lamina Propria Dissociation Kit (Miltenyi Biotec).

Measurement of cytokines in tissue homogenization

Cytokines in serum were measured by BD Cytometric Bead Array (CBA) Kit (BD Biosciences, San Jose, CA). Serum IL-2 was measured by BD mouse IL-2 ELISA Kit (BD catalog# 55148).

Histological analysis

Tissue specimens were fixed in formalin before embedding in paraffin blocks, sectioned and stained with H&E. Slides were examined at 100X or 200X magnification and visualized with a Zeiss Observer II. Each segment of colon was given a score of 0–4: grade 0, no significant changes; grade 1, minimal scattered mucosal inflammatory cell infiltrates, with or without minimal epithelial hyperplasia; grade 2, mild scattered to diffuse inflammatory cell infiltrates, sometimes extending into the submucosa and associated with erosions, with mild to moderate epithelial hyperplasia and mild to moderate mucin depletion from goblet cells; grade 3, moderate inflammatory cell infiltrates that were sometimes transmural, with moderate to severe epithelial hyperplasia and mucin depletion; grade 4, marked inflammatory cell infiltrates that were often transmural and associated with crypt abscesses and occasional ulceration, with marked epithelial hyperplasia, mucin depletion, and loss of intestinal glands. For Paneth cell quantification, a total of 9 pictures from 3 different locations of the H&E-stained slides from one mouse were taken under 200 x magnification, and total Paneth cell and crypt numbers were counted, Paneth cell numbers per crypt are shown.

Imaging mess cytometry (IMC) and Immunofluorescence Staining

1. Sample preparation and staining: Formalin-fixed, paraffin-embedded (FFPE) tissue slides were deparaffinized with xylene for three cycles of 10 minutes each. The sections were subsequently rehydrated through a graded ethanol series (two cycles at 100%, followed by one cycle each at 90%, 80%, and 65%) for 5 minutes per step, and finally immersed in Milli-Q water for 5 minutes. Antigen retrieval was performed using IHC Antigen Retrieval Solution (10X High pH, eBioscience) in a decloaking chamber at 95°C for 65 minutes. After cooling to room temperature, slides were permeabilized with 0.2% Triton X-100 in PBS for 15 minutes, followed by blocking with SuperBlock™ T20 (PBS) Blocking Buffer (Thermo) for 40 minutes. Slides were then washed briefly with PBS and incubated overnight with primary antibodies at 4°C.

Subsequently, slides underwent three washes with wash buffer (0.2% Tween 20, 0.1% BSA in PBS), each for 5 minutes.

For imaging mass cytometry (IMC), slides were incubated with an Iridium (Ir) solution (1:100 dilution) at room temperature for 10 minutes, washed twice for 5 minutes each with Milli-Q water, and air-dried immediately afterward. For immunofluorescence staining, slides were incubated with secondary antibodies for 60 minutes at room temperature, washed three times for 5 minutes with wash buffer, and then treated with TrueVIEW (Vector Labs) for 5 minutes to quench autofluorescence. After an additional wash in PBS for 5 minutes, sections were mounted with VECTASHIELD Plus mounting medium (Vector Labs).

2. Image acquisition

Images were acquired using a Hyperion Imaging System (Fluidigm). Tissues were laser-ablated at a frequency of 200 Hz. Regions containing the specimen were selected and scanned to the largest feasible area based on preliminary assessments using hematoxylin and eosin (H&E) staining and Hyperion preview images.

3. IMC data preprocessing

Raw imaging data were processed using Steinbock software (https://bodenmillergroup.github.io/steinbock/) for format conversion and cell segmentation. Subsequent dimensional reduction (t-SNE), cell type identification, and clustering analyses were performed using R and visualized using histoCAT software (Schapiro et al., 2017). Our IMC data processing workflow generally followed the methodology described in Schapiro et al. Regions expressing Ceacam1$^+$KI67$^+$ E-cadherin$^+$ were identified using QuPath software's built-in random forest classifier, validated by visual inspection. Training of the random forest model

was conducted through manual annotation of representative regions, and the resulting parameters were uniformly applied across all images.

In imaging mass cytometry (IMC), the co-localization was assessed using a quantitative approach based on single-cell segmentation. As detailed in our methods section, raw imaging data was processed using Steinbock that segments the scanned slide into single cells. This single-cell data allows for the determination of proximity and co-localization of different cell types, including T cells ($CD4^+$ and $CD8^+$) and $CD11b^+$ cells, by analyzing the spatial relationships between segmented cells. In addition, by using FIJI (ImageJ) and combining the channels for T cell and CD11b markers into one graph, we can also easily reach this conclusion visually. Therefore, our statement regarding the co-localization is based on this quantitative single-cell analysis and merge figure rather than solely on visual assessment of the provided images at that specific magnification.

For counting LPS-positive bacteria, objects smaller than 25 pixels were excluded as noise. Bacterial counts were manually quantified using ImageJ software.

16S PacBio SMRT Sequencing and data analysis

Microbial DNAs from 1-3 mg of murine fecal samples from cecum were extracted and purified according to the manufacturer’s protocol of EXT3-16S DNA Purification and PCR Amplification Kit of Shoreline Biome (Farmington, CT), and the extended (~2,500 bp) region, which contains 16S rRNA gene, the adjacent Internally Transcribed Spacer (ITS) and part of the 23S gene, was amplified using barcoded primer sets in the same kit.

For construction of SMRTbell libraries, all reagents were provided by PacBio (Menlo Park, CA) SMRTbell Template Prep Kit 1.0. Equal molar quantities of the amplicons were pooled, and 50 ul of the DNA repair mixture containing 37 ul of pooled DNA, 5 ul of DNA Damage Repair Buffer

(10X), 0.5 ul of NAD+ (100X), 5 ul of ATP high (10 mM), 0.5 ul of dNTP (10 mM) and 2 ul of DNA Damage Repair Mix were incubated at 37°C for 20 minutes. To generate blunt ends of the DNA, 2.5 ul of End Repair Mix (20X) was treated at 25°C for 5 minutes. 40 ul of the adapter ligation mixture containing 20 ul of blunted ended DNAs, 5 ul of Annealed Blunt Adapter (20 uM), 4 ul of Template Prep Buffer (10X), 2 ul of ATP low (1 mM) and 1 ul of ligase (30 U/ul) was incubated at 25°C overnight. To degrade failed ligation products, 1 ul of ExoIII (100.0 U/ul) and ExoVII (10.0 U/ul) was treated to the mixture at 37°C for 1 hour.

To produce polymerase complexes, all reagents were provided by PacBio (Menlo Park, CA) and the reagent concentrations in the protocol were calculated by the Sample Setup module in PacBio SMRT Link (v8.0.0.80529). The Sequencing Primer v2 (1 ul) was diluted by 29 ul of Elution Buffer and the mixture was incubated at 80°C for 2 minutes. And, the conditioned sequencing primers were incubated with the library at 20°C for 1 hour. 1 ul of Sequel Polymerase 3.0 was diluted by 9 ul of Sequel Binding Buffer, first. And, the polymerase was further diluted by adding 1 ul of Sequel Binding Buffer into 3.2 ul of diluted polymerase. The diluted polymerase was applied to the library with the sequencing primers, and the mixture was incubated at 30°C for 1 hour. After purification of the polymerase complexes using AMPure PB Beads, 85 ul of the final loading dilution in the sample plate was loaded into Sequel. The concentration of the sample on the plate was 8 pM and Movie Time was 10 hours and 2 hours of Pre-Extension Time was applied. The primary analyses, including real-time signal processing and base calling were processed by a built-in PacBio Blade Center through Sequel ICS, and result stream directly to SMRT Link (v8.0.0.80529). The CCS reads (> 5 Minimum Number of Passes and 0.99 Minimum Predicted Accuracy) were produced by the Circular Consensus Sequences (CCS) module in SMRT Link (v8.0.0.80529). The demultiplexing and the taxonomic classification analysis of the CCS reads were carried out using SBanalyzer (v2.4-2) of Shoreline Biome (Farmington, CT) based on Athena V2 database.

Single-cell sequencing library construction using the 10x Genomics Chromium Platform

Library preparation was done with the Chromium Single Cell 5' Reagent Kits from 10× Genomics according to manufacturer's protocol. Cellular suspensions were loaded on a Chromium Controller instrument (10x Genomics) to generate single-cell gel bead-in-emulsions (GEMs). GEM-reverse transcriptions (GEM-RTs) were performed in a Veriti 96-well thermal cycler (Thermo Fisher Scientific). After RT, GEMs were harvested, and the cDNAs were amplified and cleaned with the SPRIselect Reagent Kit (Beckman Coulter). Indexed sequencing libraries were constructed using the Chromium Single-Cell 5′ Library Kit (10x Genomics) for enzymatic fragmentation, end-repair, A-tailing, adaptor ligation, ligation cleanup, sample index PCR, and PCR cleanup. The purity and library size were validated by capillary electrophoresis using 2,100 Bioanalyzer (Agilent Technologies). The quantity was measured fluorometrically using Qubit dsDNA HS Assay Kit from Invitrogen.

Libraries were sequenced with a NovaSeq 6000 instrument (Illumina) to a depth of 35k-40k reads per cell. Raw sequencing data were processed using the 10x Genomics' Cell Ranger pipeline (version 3.1.0) to generate FASTQ files and aligned to mm10 genome to generate gene expression counts. The subsequent data analysis was performed using "Seurat v3.0" package and R scripts. Cells with mitochondrial read > 10% and < 200 detectable genes were considered as low-quality and filtered out. Normalized and scaled data were clustered using the top significant principal components of 2000 highly variable genes and resolution of 0.4 using "Seurat". The t-distributed stochastic neighbor embedding (t-SNE) algorithm was used to visualize the resulting clusters. Cluster specific markers were identified using "Seurat" to generate the heatmap of marker genes in these cell clusters. Genes were compared between different clusters using Bioconductor package "Limma" and log2 normalized data. Gene Set Enrichment analysis (GSEA) v3 was performed using genes ranked by the –log10 of "Limma"

comparison P value to evaluate the significant activation or inhibition of the Hallmark gene sets in MSigDb (https://www.gsea-msigdb.org/gsea/msigdb/genesets.jsp?collection=H). All plots were generated using either “Seurat” or “ggplot2” package in R.

Reference

1. Chakraverty R, Cote D, Buchli J, et al. An inflammatory checkpoint regulates recruitment of graft-versus-host reactive T cells to peripheral tissues. *J Exp Med*. 2006;203(8):2021-2031.

Supplementary Table 1. Imaging-mass cytometry antibody panel

	Tag	Antigen	Clone	Supplier	Product ID	Final dilution
1	148Nd	Pan-Keratin	C11	Fluidigm	3148020D	1:100
2	149Sm	CD11b	EPR1344	Fluidigm	3149028D	1:50
3	156Gd	CD4	EPR6855	Fluidigm	3156033D	1:50
4	158Gd	E-Cadherin	24E10	Fluidigm	3158029D	1:100
5	162Dy	CD8a	C8/144B	Fluidigm	3162034D	1:50
6	166Er	CD45RA	HI100	Fluidigm	3166031D	1:100
7	167Er	Granzyme B	EPR20129-217	Fluidigm	3167021D	1:100
8	168Er	Ki-67	B56	Fluidigm	3168022D	1:50
9	169Tm	Collagen Type I	Polyclonal	Fluidigm	3169023D	1:100
10	170Er	CD3	Polyclonal	Fluidigm	3170019D	1:50
11	173Yb	CD45RO	UCHL1	Fluidigm	3173016D	1:100
12	191Ir/193Ir	DAPI	NA	Fluidigm	201192A	1:200
13(Self conjugated	144Nd	T-bet	4B10	Biolegend **Fluidigm**	644825 201144A	1:25
14(Self conjugated	145Nd	AHR	RPT9	Invitrogen Fluidigm	MA1-513 201145A	1:50
15(Self conjugated)	152Sm	IL-22	Polyclonal	R&D systems, Fluidigm	AF782, 201152A	1:25
16(Self conjugated	153Eu	IFN-γ	Polyclonal	R&D systems, Fluidigm	AF-285-NA, 201153A	1:25
17(Self conjugated	163Dy	IL-17A	Polyclonal	R&D systems, Fluidigm	AF-317-NA, 201163A	1:50
18(Self conjugated	175Lu	Ceacam-1	Polyclonal	R&D systems, Fluidigm	AF6480 201175A	1:100
